# Identification of Active Compounds against Melanoma Growth by Virtual Screening for Non-Classical Human DHFR Inhibitors

**DOI:** 10.3390/ijms232213946

**Published:** 2022-11-11

**Authors:** Andrés Felipe Vásquez, Luis Alberto Gómez, Andrés González Barrios, Diego M. Riaño-Pachón

**Affiliations:** 1 Grupo de Diseño de Productos y Procesos (GDPP), School of Chemical Engineering, Universidad de los Andes, Bogotá 111711, Colombia; 2 Naturalius SAS, Bogotá 110221, Colombia; 3 Laboratorio de Fisiología Molecular, Instituto Nacional de Salud, Bogotá 111321, Colombia; 4 Department of Physiological Sciences, School of Medicine, Universidad Nacional de Colombia, Bogotá 11001, Colombia; 5 Laboratório de Biologia Computacional, Evolutiva e de Sistemas, Centro de Energia Nuclear na Agricultura (CENA), Universidade de São Paulo, Piracicaba 05508-060, SP, Brazil

**Keywords:** DHFR inhibitors, pharmacophore modeling, molecular docking simulations, virtual screening, melanoma, drug screening assays, drug discovery

## Abstract

Antifolates such as methotrexate (MTX) have been largely known as anticancer agents because of their role in blocking nucleic acid synthesis and cell proliferation. Their mechanism of action lies in their ability to inhibit enzymes involved in the folic acid cycle, especially human dihydrofolate reductase (hDHFR). However, most of them have a classical structure that has proven ineffective against melanoma, and, therefore, inhibitors with a non-classical lipophilic structure are increasingly becoming an attractive alternative to circumvent this clinical resistance. In this study, we conducted a protocol combining virtual screening (VS) and cell-based assays to identify new potential non-classical hDHFR inhibitors. Among 173 hit compounds identified (average logP = 3.68; average MW = 378.34 Da), two—herein, called C1 and C2—exhibited activity against melanoma cell lines B16 and A375 by MTT and Trypan-Blue assays. C1 showed cell growth arrest (39% and 56%) and C2 showed potent cytotoxic activity (77% and 51%) in a dose-dependent manner. The effects of C2 on A375 cell viability were greater than MTX (98% vs 60%) at equivalent concentrations and times. Our results indicate that the integrated in silico/in vitro approach provided a benchmark to identify novel promising non-classical DHFR inhibitors showing activity against melanoma cells.

## 1. Introduction

**Figure 1:**
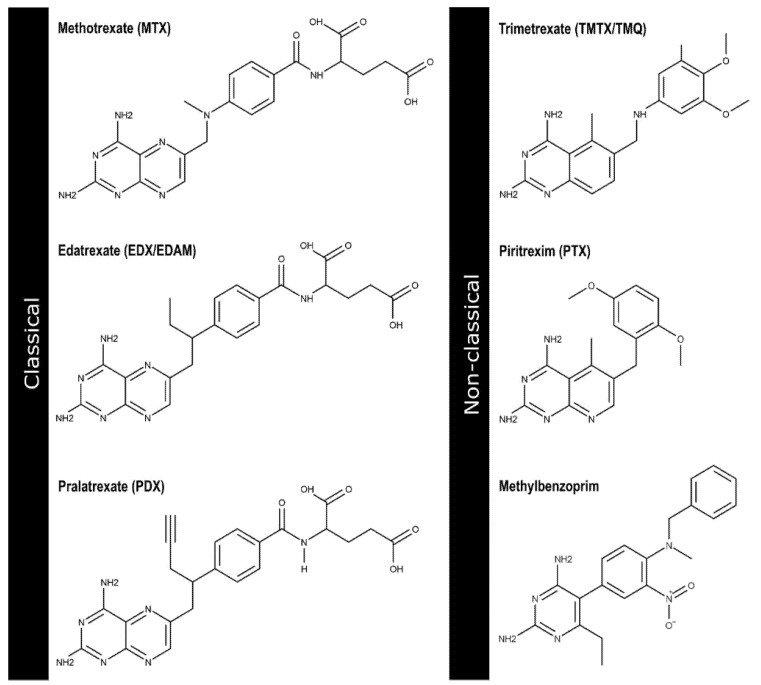
Classical and non-classical human DHFR inhibitors. The 2D chemical structures of methotrexate (ZINC01529323), edatrexate (ZINC01618702), pralatrexate (ZINC01536109), trimetrexate (ZINC00598852), piritrexim (ZINC00000640), and methylbenzoprim (ZINC03777839) were prepared with MarvinSketch v17.14, 2017, ChemAxon.

Antifolates, one of the earliest drugs for treating cancer, are known to target the folic acid cycle metabolism, in particular by inhibiting human dihydrofolate reductase (hDHFR) [1,2]. This is an NADPH-dependent enzyme responsible for the conversion of dihydrofolate (DHF) to tetrahydrofolate (THF), which is essential for DNA synthesis and cell proliferation [3,4,5,6]. Classical antifolates as the well-known DHFR inhibitors methotrexate (MTX) and aminopterin (AMT) possess a p-aminobenzoylglutamate side-chain that enables them to use the reduced folate carrier (RFC) and/or folate receptor (FR) transport systems to enter into cell and be metabolized by the enzyme folylpolyglutamate synthetase (FPGS) into poly(γ-glutamates) to ensure their cellular retention [7,8]. However, although the ‘classical’ compounds have been successfully used in a broad variety of cancer types [9,10,11], they are ineffective in melanoma—-a lethal skin malignant tumor—precisely because of their impaired cellular uptake and inadequate rate of polyglutamation, among other resistance-associated mechanisms [12,13]. This fact represents a daunting challenge, especially considering the public health threat that melanoma constitutes: it is highly metastatic, difficult to diagnose, and has had a dramatically increasing incidence over the last half-century compared with other cancers [14,15,16]. In this context, an alternative type of inhibitors known as ‘non-classical’ [17,18] could be used as a more helpful strategy. Since they exhibit a lipophilic structure instead of the classical glutamate chain (Figure 1), they may enter tumor cells by passive diffusion and not depend on their need for polyglutamation [5,19]. Therefore, the identification of novel ‘non-classical’ inhibitors represents an attractive therapeutic approach to circumvent melanoma-specific resistance while still maintaining a high affinity for their primary target.

Virtual screening (VS) has proven an effective strategy to discover novel drugs against melanoma—mong many other cancer types—by facilitating the evaluation of large collections of small molecules and identifying candidates capable of binding to a clinical target of interest [20,21,22,23]. Three approaches have become increasingly common in VS: pharmacophore modeling, molecular docking, and molecular dynamics (MD) simulations. Pharmacophore modeling is based on an abstract description of the physicochemical features (typically shared by biologically active molecules) necessary for the interaction with a target structure [24,25,26], while docking focuses on an estimation of both the preferred pose adopted by a ligand when bound to a receptor and its corresponding binding energy [27,28,29]. By performing MD simulations, we gained a comprehensive understanding of the role of key residues in the interaction between proteins and ligands as well as the conformational dynamics of a protein in bound or unbound forms [30,31,32]. However, and most importantly, a combination of pharmacophore modeling, docking, and MD simulations has proven successful in VS campaigns during the last few years: it contributes to reducing both the required computational effort and the number of false positives, providing a promising or ‘elite’ set of hit compounds that may be worth considering to submit to an eventual experimental validation [33,34,35].

The aim of this study was to identify virtual candidates of ‘non-classical’ hDHFR inhibitors potentially active against melanoma growth. We developed a pharmacophore- and docking-based VS protocol to obtain a set of ‘non-classical’ hits, two of which showed cytostatic (C1) and cytotoxic (C2) effects on human A375 and mouse B16 melanoma cells. This is the first report of potential hDHFR inhibitors derived from the combination of both VS approaches.

## 2. Results

### 2.1. Generation and Validation of Pharmacophore Models and Preliminary Screening

**Figure 2:**
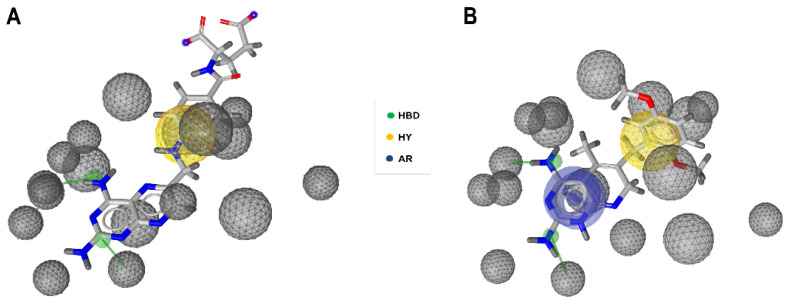
Pharmacophore models generated by the software LigandScout. Overlay of pharmacophore models (**A**) Pharm-A and (**B**) Pharm-B with the well-known active compounds aminopterin (AMT) and piritrexim (PTX), respectively. Both of these compounds were used in the decoy set. Pharmacophore features are colored as follows: hydrogen bond donor—HBD (green), hydrophobic group—HY (gold), and aromatic ring—AR (blue). Gray spheres represent excluded volume. The two H-bond donor groups point to hDHFR active site residues Ile7 and Glu30 respectively.

Two hypotheses, called Pharm-A and Pharm-B, were generated. Pharm-A consisted of two HBD features pointed towards hDHFR active site residues Ile7 and Glu30, respectively, one HY feature, and seventeen exclusion volume spheres (Figure 2A). Pharm-B described the same features as Pharm-A but also included an aromatic ring (AR) feature from the pyrimidine moiety (Figure 2B). The crystal 3D structure used for the generation of both pharmacophore models included interaction with the most important hDHFR amino acid residues (Table S1). Pharm-B model exhibited better accuracy, specificity, and AUC/EF values compared to Pharm-A, especially at early stages (Figure S1 and Table S2). After the VS, 371 compounds (0.28% of the screened database) were retrieved.

### 2.2. Ensemble Generation and Docking-Based Screening

After the alignment of 54 native co-crystal hDHFR structures and the evaluation of Dunbrack, Dynameomics, and Richardson rotamers for the key active site residue Phe31, we selected three structures to generate a conformational ensemble. These crystal structures (PDB codes: 1KMV [36], 1U72 [37], and 3NXV [38]) which described a side-chain conformation very similar to the rotamers with the highest probability (Figure S2) were used for docking procedures. Consequently, we retrieved a final hit dataset of 173 compounds.

### 2.3. Clustering and Selection of Hit Compounds C1 and C2

As shown in Table 1, the average molecular weight and logP of this hit dataset (after docking) were 378.34 Da and 3.68, respectively, and the estimated average number of rings was 3.42 (2.55 for aromatic rings). On the other hand, hierarchical and multidimensional clustering revealed a moderately high distribution for hit compounds (Figure S3). We selected a couple of hits named C1 (ZINC00907702; 2-(4,6-diaminopyrimidine-1,3-diium-2-yl)sulfanyl-*N*,*N*-diphenylacetamide and C2 (ZINC20102709; *N*-[3-[[2-(4,6-diaminopyrimidin-2-yl)sulfanylacetyl]amino]phenyl]adamantane-1-carboxamide) for biological evaluation (Figure S4), which were found in different clades or regions according to the clustering (Tanimoto index: 0.28). The specific MW, logP, and the number of rings of these compounds are listed in Table 1. According to the prediction results by the web tool Property Explorer, C1 and C2 exhibited no apparent toxicological molecular properties.

### 2.4. Re-Docking and Analysis of Binding Mode and Energy of C1 and C2

**Figure 3:**
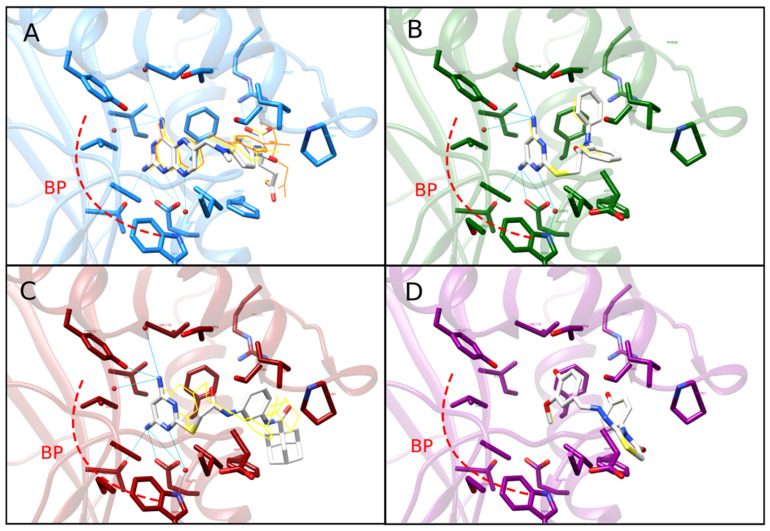
Predicted binding mode of compounds C1 and C2 on human DHFR active site. Superimposed 3D docked poses of the inhibitor MTX (**A**), hit compounds C1 (**B**) and C2 (**C**), and an hDHFR decoy (**D**) interacting with the hDHFR active site. In each case, ligands from initial docking are displayed in a yellow wire representation, and those from re-docking (i.e., including a structurally conserved water molecule) are shown as white sticks colored by heteroatom. The structure of hDHFR is represented as ribbons and the side chains of critical active site residues are shown as sticks colored by heteroatom. The original pose for MTX (PDB code: 1U72) is represented as an orange wire (see panel (**A**)). H-bonds are shown only for re-docking poses and are displayed as blue continuous lines. The main binding pocket (BP site) of DHFR is illustrated in all panels as a red discontinuous curved line.

The analysis via Relibase allowed the identifying of one structurally conserved water molecule (HOH302 in the hDHFR co-crystal structure with the best resolution—PDB ID: 1KMV). Hence, this molecule was included in a re-docking procedure. We found that the predicted binding energy for all evaluated compounds (C1, C2, MTX, and hDHFR decoy) were similar to those obtained after the first docking protocol (Table S3). However, as expected, the binding energy for C1 and C2 was predicted to be better than that for the hDHFR decoy (though not as good as that for MTX). H-bonding of C1 and C2 with various critical binding pockets (herein, called BP site) residues such as Glu30 and Ile7 was observed after docking (Figure 3), which was in good agreement with the analysis resulting from the overlay of C1 and C2 with Pharm-B model (Figure S5). Additionally, we observed that the included water molecule mediated interaction between either C1 or C2 and the hDHFR binding site residues Glu30 and Trp24, which was also observed for MTX (both in docking pose and x-ray crystal structure), but not for the hDHFR decoy (Figure 3). Hydrogen bonding with Trp24 was observed only after re-docking. Interestingly, hit compound C2 adopted a conformation known as ‘flip mode’ after the re-docking including this water molecule. A dramatic pucker in the diaminopyrimidine ring of C1 and C2 was also observed. Pyrimidinyl and diphenylacetamide rings of C1 as well as pyrimidinyl and aminophenyl rings (and adamantane-1-carboxamide moiety) of C2 were predicted to establish hydrophobic contacts with Val8, Ala9, Leu22, Phe31, Phe34, Ile60, and Leu67 in the same pocket (Figure S6). A good agreement was observed between these results and those obtained after using FlexX (Figure S7).

### 2.5. Molecular Dynamics Simulations

**Figure 4:**
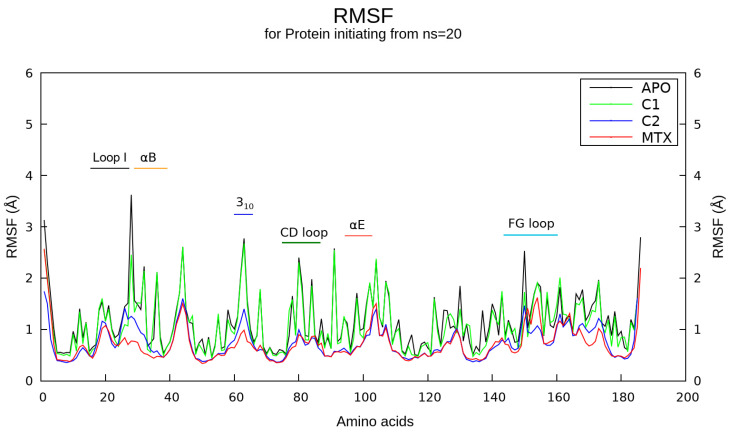
Average RMSF plot for hDHFR in a bound and unbound form. The per-residue RMSF calculation for the free or unbound form, as well as the bound form complexed with C1, C2, and MTX, are depicted by continuous lines in a black, green, blue, and red color, respectively. Specific secondary structures are reported at the top of the graph.

The resulting root-mean-square deviation (RMSD) for the backbone atoms of DHFR (Figure S8) rapidly converged around 1.5 Å throughout most of the time course of the simulations, especially after the first 20 ns. All protein-ligand complexes proved to have RMSD values similar to the unbound form of hDHFR. We also analyzed the per-residue root mean square fluctuation (RMSF) data to analyze differences between the systems, observing notable fluctuations around residues 27–30 (reaching a peak around 3.8 Å) which marks the end of Loop I and the beginning of the helix-αB (Figure 4). This region, observed to be highly flexible in the unbound form of hDHFR, was shown to gain more rigidness once the protein was in complex with any of the evaluated ligands, although this rigidity was slightly lower for C1. On the other hand, a 3_10_ helix near to helix-αC, helix-αE, CD loop, and FG loop (these three latter according to the numbering stated in [39]) were shown to be more rigid once the enzyme was complexed with any compound of the study compared to the unbound (free) form. It was interesting to note that several binding site residues experienced major fluctuations upon binding of ligands (particularly, for MTX and C2) in comparison to the unbound system The hit compound C1 showed an RMSF profile very similar to the unbound system except the border between Loop I and helix-αB (both part in active site) and the FG loop (distal region of active site). The potential energy and kinetic energy for hDHFR showed a fairly constant value (Figure S9 and S10, respectively), which was in line with the total energy for both bound and unbound forms (Figure S11). Volume plots indicated a constant value for the production phase of the simulations (Figure S12). After quickly reaching the target value (300 K) during the heating phase, the temperature of the system remained stable throughout the production phase (Supplementary Materials Figure S13). Finally, during the 250-ns production running, the pressure and density of each protein and protein-ligand system changed only slightly, as expected (Figures S14 and S15, respectively).

### 2.6. Binding Free Energy and DFT Analyses

The MM-PBSA results indicated favorable ∆G binding values for compounds C1 and C2, although they were smaller compared to the reference active compound MTX (Supplementary Materials Figure S16 and Table S4). Several residues implicated in the binding energy contribution for the hit compounds C1 and C2 were also observed for MTX, including Ile7, Phe31, Phe34, and Ile60. However, Leu22 and Pro61 were not observed for MTX, and Arg32 (very important for MTX) was present in C2 but not in C1. Arg70, as expected, was observed for none of the hit compounds C1 or C2, but it accounted for a large energy contribution in MTX. Depending on the particularly evaluated protein–ligand complex, these residues accounted for more than 3 kcal/mol contribution in ∆G binding (Supplementary Materials Figure S17). As a further validation, electronic parameters for MTX, C1, and C2 were reported according to DFT calculations. Interestingly, the energy gap values of C1 and C2, based on HOMO and LUMO calculated values were observed not only to be similar to each other but also proved to be considerably higher (+50% approximately) compared to MTX (Table S4).

### 2.7. ADME Profiling

On the other hand, the prediction of ADME properties provided complementary results (Table S5). In contrast to MTX and C2, the hit compound C1 was predicted to inhibit cytochrome isoforms CYP1A2, CYP2C9, and CYP3A4. However, it was also predicted as a non-Pgp substrate estimated with a high GI absorption level and a much lower synthetic accessibility score compared to the other evaluated compounds. On the other hand, C2 was predicted as a non-inhibitor of the inhibit cytochrome isoforms (excepting CYP3A4) but, similarly to MTX, it was estimated as a Pgp substrate with a low GI absorption level, and a considerably higher synthetic accessibility score. C1 successfully satisfied all the different evaluated drug-likeness scores, whereas C2 and MTX had one violation of Veber, Egan, and Muegge rules in terms of their corresponding topological polar surface area (TPSA) values. C1, C2, and MTX were all predicted as non-BBB permeants that showed no structural alerts in the base of both PAINS and Brenk rules. Remarkably, the bioavailability score for C1 and C2 was dramatically higher (5 times greater) compared to MTX.

### 2.8. Biological Assay on Melanoma Cells

**Figure 5:**
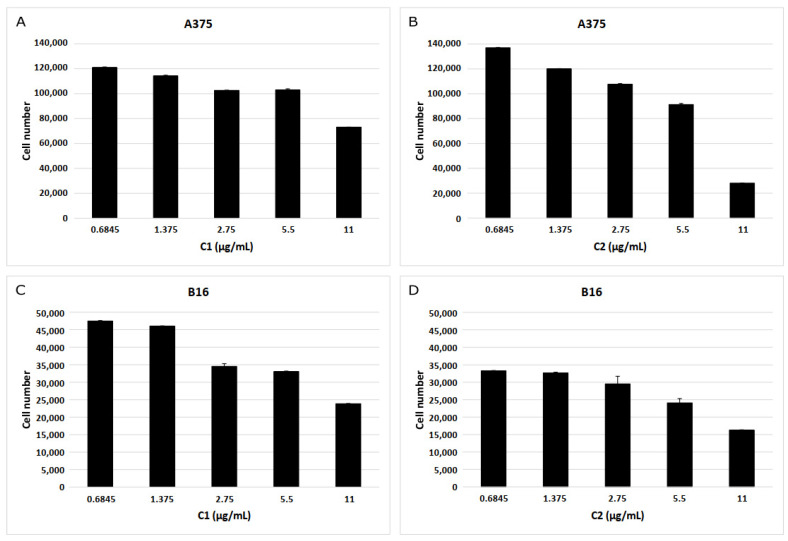
MTT reductase activity of human and mouse melanoma cells after exposure to C1 and C2. Both human (A375, upper panels) and mouse (B16, lower panels) melanoma cells were exposed to variable concentrations of hit compounds C1 (**A**,**B**) and C2 (**C**,**D**) for 48 h.

**Figure 6:**
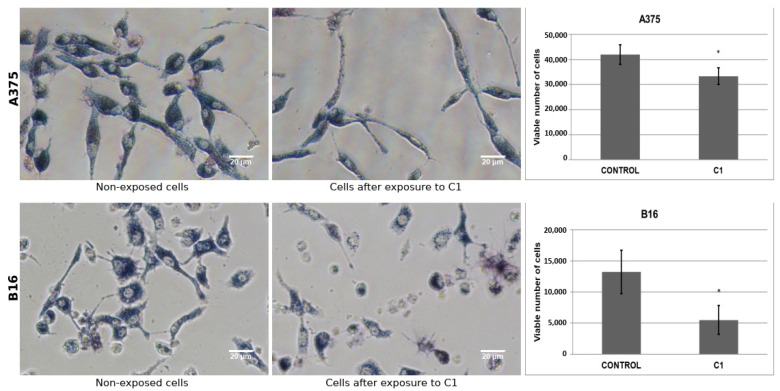
Effect of compound C1 on human and mouse melanoma cell lines. Human (A375, upper panels) and mouse (B16, lower panels) melanoma cells were seeded into a 48-well plate overnight and treated with C1 for 48 h. Cell viability was determined by MTT reductase activity (photographs) and trypan blue (TB) exclusion assay (bar graphs) after exposure to hit compound C1 (shown here at a concentration of 11 µg/mL for human cells and 2.75 µg/mL for mouse cells). * Statistically significant.

**Figure 7:**
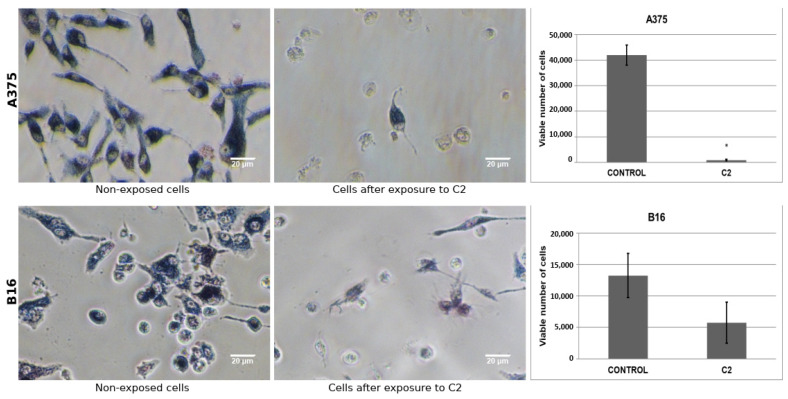
Effect of compound C2 on human and mouse melanoma cell lines. Human (A375, upper panels) and mouse (B16, lower panels) melanoma cells were seeded into a 48-well plate overnight and treated with C2 for 48 h. Cell viability was determined by MTT reductase activity (photographs) and trypan blue (TB) exclusion assay (bar graphs) after exposure to hit compound C2 (shown here at a concentration of 11 µg/mL for human cells and 2.75 µg/mL for mouse cells). * Statistically significant.

Following the selection and bioinformatics analysis of C1 and C2, these hit compounds were subjected to biological assays on melanoma cells. In accordance with calibration curves, linear relationships between formazan absorbance (OD 570 nm) and cell number for both A375 (R2 = 0.9998) and B16 (R2 = 0.9995) cell lines were obtained (Figure S18). MTT reductase activity of melanoma cell lines decreased after exposure to increasing concentrations of compounds C1 and C2. On average, for C1, cell viability on human and mouse cells at 11 µg/mL was decreased by 39 ± 0.3% and 56 ± 0.2%, correspondingly, compared to non-exposed cells (control group) (Figure 5). Likewise, for C2, on average, cell viability on human and mouse cells at 11 µg/mL decreased 77 ± 0.2% and 51 ± 1.0%, respectively, compared to unexposed cells. As shown in Figure 5 and Figure 6, MTT reductase activity decreased after human and mouse cells exposure to C1 or C2 for 48 h, compared to non-exposed cells. On the other hand, we observed a reduction in the number of cells excluding trypan blue dye indicating a loss of cell viability. For C1, there was a decrease of 21 ± 8.1% for 58 ± 17.5% for human (11 µg/mL) and mouse (2.75 µg/mL) cells (Figure 6), respectively, compared to unexposed cells. In comparison, for C2, a decrease of 98 ± 1.0% and 57 ± 25.0% was observed for human (11 µg/mL) and mouse (2.75 µg/mL) cells (Figure 7), correspondingly, in comparison to unexposed cells. The LC50 value for C1 was estimated to be 15.6 ± 1.1 µg/mL (44.39 ± 2.56 µM) and 8.6 ± 0.6 µg/mL (24.47 ± 1.71 µM) for human and mouse cell lines, respectively. Likewise, the estimated LC50 value for C2 was 7.9 ± 0.7 µg/mL (17.45 ± 1.55 µM) and 11.0 ± 0.5 µg/mL (24.30 ± 1.10 µM) for human and mouse cell lines, correspondingly.

We observed a reduction of MTT reductase activity in both human and mouse melanoma cell lines after increasing exposure times to C1 or C2 (Figure S19). On average, cell viability on human (A375) melanoma cells after an exposition period of 24 h (at 11 µg/mL) was observed to decrease 36 ± 1% and 49 ± 2% for C1 and C2, respectively. However, for C1, the cell number was recovered at 36 and 48 h. In contrast, exposure to C2 induced much lower values than those observed at later times (66 ± 2% at 36h and 78 ± 3% at 48 h). We obtained similar results for mouse melanoma cells. As shown in Figure S20, after the exposition to compound C1 at a concentration of 11µg/mL for 48 h, the number of cells that excluded Trypan Blue dye declined in a similar but lower degree compared with exposition to either MTX (21 ± 6% vs. 60 ± 12%) or AMT (20 ± 8% vs. 58 ± 10%), in all cases having non-exposed cells as a frame of reference. In contrast, after exposition to C2 at equal concentration and time, the human cell number declined much more dramatically (98 ± 2%) compared with exposition to MTX and AMT, and C1.

## 3. Discussion

This research work aimed to identify novel potential ‘non-classical’ hDHFR inhibitors by a pharmacophore- and ensemble docking-based VS and to assess the effect of two of them on the viability of human and mouse melanoma cells. To our knowledge, this is the first report combining both VS approaches as a strategy to identify virtual candidates of this enzyme, which represents a contribution to the search for new compounds against hDHFR and melanoma.

Although model PharmA showed a slightly higher sensitivity after ROC curve analysis, Pharm-B was demonstrated to be a more appropriate query to use in the VS, because it showed higher specificity, accuracy, and AUC/EF values, especially at early stages. Certainly, high early AUC/EF values and low false positive rates—even at the risk of losing some potential active molecules—have been highlighted as critical decision-making criteria in VS campaigns [40,41,42], especially considering the eventual biological assays which hit compounds are frequently submitted to. Furthermore, the AR feature present in Pharm-B (but not in Pharm-A) facilitated the interaction with a greater number of critical active site hDHFR residues and provided a “puckering” associated with an h-bond geometry advantageous for hDHFR binding [36]. On the other hand, the large differences among the side-chain angles of Phe31 rotamers corroborate the remarkable flexibility of this hDHFR active site residue described in former reports [43,44,45] and, by extension, suggests that the use of an ensemble-docking strategy was appropriate to handle conformational changes in this particular biological system.

Clustering and assessment of the physicochemical properties of compounds in the hit dataset were critical to evaluate the performance of the VS strategy. The average MW and logP of the hits indicate a great potential for the development of these compounds, considering that these features usually increase during eventual hit optimization [46] and more than three aromatic rings per molecule could be a disadvantage [47]. On the other hand, the significant chemotype diversity showed by the hit dataset indicates that the general VS approach was successful in providing numerous different starting points (molecular diversity), as recommended by [48], and validates the selection of C1 and C2 as representatives from different clusters. We did not observe large differences in the binding pose of C1 and C2 after re-docking procedures. However, the included water molecule was observed to be involved in h-bonds involving Glu30 and Trp24, indicating a role of this water molecule in mediating critical protein-ligand interactions. Strikingly, key differences observed for the C2 binding pose after the inclusion of this water molecule suggest a role in the so-called ‘flip’ mode (which implies a 180° rotation about the C-2, NH2 bond) which has been previously observed for MTX and other antifolates [49]. The h-bonds were observed for MTX but not for the hDHFR decoy, and the similar results obtained by FlexX further supported these results.

According to the backbone RMSD profile data obtained from the MD simulations, the enzyme hDHFR in its free and bound states is generally structurally stable. Although MMPBSA data showed stronger binding energy for MTX compared to C1 and C2, the corresponding binding energies of both hit compounds, very similar to each other, indicated that our VS procedure helped identify promising compounds, particularly considering three facts. First, although we used MTX as a reference compound since it is a well-known classical hDHFR inhibitor and one of the drugs in the World Health Organization’s list of essential medicines [50], a comparative framework of affinity with other compounds (especially in development phase) is not necessarily intuitive because this drug exhibits a very strong activity that achieves even low picomolar range concentrations [51]. Second, it is worth mentioning that during the hit-discovery stage we develop through this study, compounds are typically obtained in the micromolar range and are only improved during optimization [52,53]. Finally, and most important, it must be considered that the substitution of a glutamate moiety present in classical antifolates such as MTX for a lipophilic moiety in C1 and C2 was expected to eliminate the salt bridge we observed between MTX and hDHFR Arg70 in hDHFR and, therefore, dramatically diminish the predicted affinity of both hit compounds. Interestingly, the larger energy gaps observed for C1 and C2 compared to MTX suggest that these hit compounds have a low chemical reactivity and high kinetic stability, increasing their potential to be further optimized and analyzed in terms of their bioactivity [54,55]. On the other hand, the RMSF analyses for hDHFR indicate certain key roles played by regions such as Loop I, helix-αB, and a short helix 3_10_ near helix-αC. These regions, observed to be more rigid in the bound form of the enzyme in comparison with the unbound form, have been reported in recent studies and might pinpoint specific residues into the active site of hDHFR fluctuate to accommodate these compounds in the binding site [56,57]. Interestingly, the large Loop I has been reported to include a left-handed polyproline-type helix only observed in vertebrate versions of this enzyme [44]. We believe that these results are in good agreement with the energy contribution of hDHFR residues positively taking part of the mentioned regions such as Phe31, Leu22, and Pro61, the two latter proving relevant in the energy contribution of protein–ligand interaction for C1 and C2 but not for MTX. Phe31 has long been considered a gatekeeper residue for hDHFR (see Methods Section 4.4) whereas Pro61 might account for critical interactions with aromatic moieties in both hit compounds, as has been indicated by a number of studies during the last two decades [58,59,60,61]. As a complementary result, it is intriguing to notice the promising ADME profile of both hit compounds C1 and C2, from which should be highlighted their large predicted bioavailability despite their lipophilicity (in comparison to MTX), a highly discussed topic in recent years [62,63].

MTT reductase activity allowed us to evaluate the effect on the growth and viability of human and melanoma cells after exposure to hit compounds C1 and C2. The similar decreasing trend in cell viability that we observed for C1 compared to reference compounds MTX and AMT, as well as the recovery of cell growth at 36 and 48 h, indicated a reversible cytostatic effect. This finding is in line with previous reports for MTX that support this type of mechanism on mouse melanoma cell line B16 [13]. Likewise, the most remarkable decrease in cell viability caused by C2 compared to MTX and AMT suggested a significant cytotoxic activity. Intriguingly, this effect might be explained by its adamantyl moiety, which has been previously reported for other highly potent anti-cancer compounds [64,65]. The direct count of cells excluding trypan blue (TP) represented an additional benchmarking to confirm these results. It will be valuable, however, to conduct further studies giving insight into the inhibitory activity of these hit compounds and providing a better understanding of their respective mechanisms of action mainly because of two different facts. On the one hand, several “non-classical” antifolates have been demonstrated as effective not only against other cancer types different from melanoma [66,67] but also against infections caused by either bacteria [68] or parasites from Trypanosoma or Plasmodium genres [19,69,70]. On the other hand, several antifolates have been observed to be active against multiple enzymes in the folic acid cycle apart from DHFR such as thymidylate synthase (TS), GAR formyltransferase (GARFT), or AICAR formyltransferase (AICARFT) [51,71], reason by which we believe that C1 and C2 (or a series of analogues) might be part of a multi-targeted strategy intended to block simultaneously two or more enzymes within the same metabolic pathway. 

## 4. Materials and Methods

**Figure 8:**
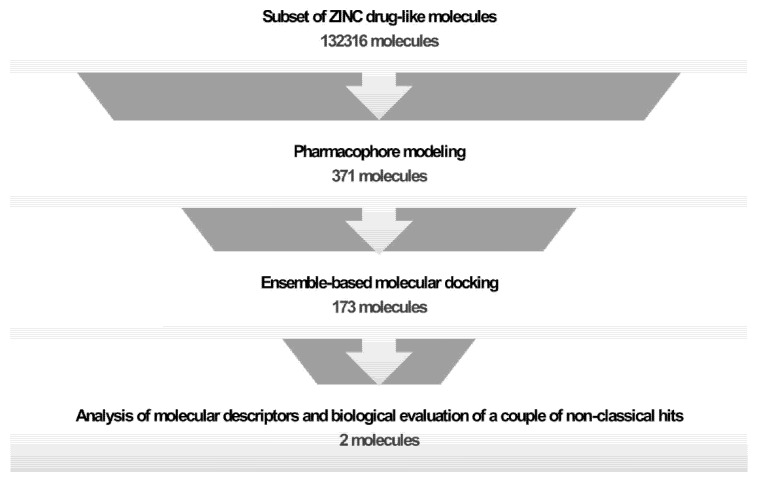
Schematic representation of the steps along the virtual high-throughput screening (vHTS) process. The general strategy involved the arbitrary selection of a set of drug-like molecules retrieved from the ZINC database, the combination of pharmacophore modeling and docking procedures, the analysis of molecular descriptors (MW, LogP, Jaccard–Tanimoto coefficient), and the biological evaluation of two non-classical hit compounds.

The general approach involved both computational and wet-lab experiments for selecting and evaluating a couple of non-classical lipophilic hit compounds. The strategy is outlined in Figure 8.

### 4.1. Pharmacophore Models Generation

Pharmacophore modeling was developed using Ligand Scout v3.0 [72]. As input, we employed the X-ray crystallographic structure of human DHFR in complex with cofactor NADPH and the competitive inhibitor SRI-9662 (LII; PDB code: 1KMV [36]). This ternary complex was selected because of its high near-atomic resolution (1.05 Å; see Table S4) and because of the non-classical lipophilic character of the inhibitor. We considered H-bond donor (HBD), H-bond acceptor (HBA), Negative ionizable (NI), positive ionizable (PI), Hydrophobic (HY), and Aromatic Ring (AR) feature types in generating the pharmacophore models. Finally, exclusion volume spheres were also included to represent disallowed areas in the protein.

### 4.2. Validation of Pharmacophore Hypotheses

We have validated the generated pharmacophore models using two different methods: (i) the presence of chemical features critical to interact with key active site residues and (ii) a decoy set. For the first approach, we used the software UCSF Chimera v.1.11.2rc [73] to analyze 54 native co-crystal structures downloaded from the Protein Data Bank. We identified any h-bonds according to the geometric criteria described by [74] and van der Waals (vdW) overlaps only if equal or greater than 0.4 Å (allowance of 0.0 Å) according to the formula: overlapij = rVDWi + rVDWj − dij − allowanceij [75]. Any hDHFR residue implied in ligand interaction was collected and ranked according to its frequency among the different crystallographic structures. For the second method, we used a decoy set consisting of 60 hDHFR known inhibitors with submicromolar potency retrieved from the ZINC database [76,77,78] and 8144 hDHFR inactive compounds from the Directory of Useful Decoys (DUD) [79]. We calculated receiver operating characteristic (ROC) curves [40], and parameters such as accuracy, sensitivity, specificity, area under the curve (AUC), and enrichment factor (ER) were estimated (the latter two for 1%, 5%, 10%, and 100% of the database).

### 4.3. Pharmacophore-Based Virtual Screening

After validation procedures, the pharmacophore model with the best performance was used in a virtual database screening over a subset of 132,316 commercially-available drug-like compounds retrieved from the ZINC database [76,77,78]. We considered as hits only those molecules fitting all the features from the pharmacophore model. These hits were used as input for subsequent molecular docking procedures.

### 4.4. Generation of Conformational Ensemble

In order to consider the flexibility of the enzyme during docking, we employed a two-step procedure in UCSF Chimera v.1.11.2rc [73] to construct an ensemble of protein structures [80]. Initially, we developed an alignment of native co-crystal hDHFR structures downloaded from Protein Data Bank using as reference the hDHFR X-ray structure with the highest resolution available (PDB code: 1KMV [36]). Next, we examined the Dunbrack [81], Dynameomics [82], and Richardson [83] rotamers for Phe31, a residue that has been previously observed to exhibit important side-chain conformational transitions and is considered a gatekeeper residue [4]. After visual inspection and construction of a Janin plot [84], we selected three hDHFR structures with Phe31 side-chain position nearest to the rotamers with the best probability (above 5%) and retained them to generate our conformational ensemble.

### 4.5. Ensemble-Based Molecular Docking

Remarkably, several studies have shown that the performance of docking may be remarkably improved by using multiple structures to consider receptor flexibility (which is frequently referred to as ensemble docking) [85,86]. Hence, hit compounds resulting from the previous pharmacophore-based VS were docked to the generated ensemble of human DHFR structures using the program AutoDock v4.2 [87] and a modified version of the open-source bash script Docker 1.0 [88]. Protein preparation and refinement were carried out using AutoDockTools v1.5.6rc3 [89]. We considered for further analysis only those compounds with binding energy equal to or lower than a score threshold of −9 kcal/mol. These hits were ranked according to their corresponding binding energy values and classified to that 3D structure (from the ensemble) with the best of these values. We visually inspected each docking pose to verify the appropriate binding orientation of hits into the binding site of the corresponding hDHFR structure.

### 4.6. Hit Dataset Clustering and Analysis

ChemMine Web Tools [90] and FigTree [91] were used to generate and visualize a hierarchical clustering and a multidimensional scaling (MCS) of the hit dataset after docking. Molecular weight, logP, and the number of rings (including aromatic) of each compound as well as the average values for the hit dataset were calculated. Finally, we selected a couple of compounds (here referred to as C1 and C2) for biological evaluation based on these properties. Molecular similarity and potential side effects for C1 and C2 were estimated using the Jaccard-Tanimoto coefficient [92]) and the Property Explorer Applet, which is freely available at [93].

### 4.7. Re-Docking with Conserved Water Molecules and Binding Pose Comparison

The incorporation of structurally conserved water molecules able to mediate ligand–receptor interactions has been increasingly discussed in VS campaigns [94,95]. Therefore, any structurally conserved water molecules mediating ligand interactions with hDHFR were identified in this study using the database Relibase [96]. Any possibly found water molecule was maintained in the hDHFR 3D structure and employed in a re-docking of hit compounds C1 and C2 using the program Autodock v4.2. The hDHFR inhibitor methotrexate (MTX) and one inactive compound used previously in the decoy set (ZINC00000960; (*E*)-3-[(6S,6aR)-4-hydroxy-11-keto-3,6-dimethyl-5,6,6a,7-tetrahydropyrrolo [2,1-c][1,4]benzodiazepin-8) were included in docking as positive and negative controls, respectively. For comparative purposes, we used the docking program FlexX (BioSolveIT, Sankt Augustin, Germany), to automatically include and freely rotate water molecules in docking procedures [52].

### 4.8. Molecular Dynamics (MD) Simulations

The structures of hDHFR docked along with hit compounds C1 and C2 were used as starting points for MD simulations along with the protein–ligand complex of hDHFR co-crystallized with MTX playing a role as control. We used both Amber ff14SB and GAFF force fields for the protein and the ligand, respectively [97,98], and we calculated ligand charges at the Hartree–Fock (HF) level using a 6-31 G* basis set as default parameters of Gaussian 16 package [99]. Then, the MD setup and the corresponding simulations were set up using GROMACS v2018 [100,101,102]. We employed cubic boxes, solvation with explicit TIP3P water molecules [103], and a concentration of 0.15 M for the neutralization step by Na+ and Cl− ions. Energy minimization was carried out on all protein–ligand systems with 5000 steps of steepest descent [104], followed by a heating process represented in a canonical (NVT) ensemble for 100 ps. Two subsequent equilibration steps were performed in an isothermal-isobaric (NPT) ensemble (100 ps each one) using a Berendsen barostat and a modified Berendsen thermostat (V-rescale) [105,106]: in the first equilibration step, both the protein backbone and the corresponding ligand were restrained, whereas during the second equilibration step, only the corresponding ligand was restricted. The temperature was kept to 300 K with a mesh spaced for 0.16 nm, and the pressure was kept at 1 atm. Bond lengths were constrained during the simulations using the LINCS algorithm [107]. Finally, a 250 ns production simulation was carried out for all systems in an NPT ensemble (no backbone restraining) running on a Linux workstation using an Intel Xeon Gold Processor 5118 SP 2.3 GHz (3.2 GHz Turbo) with 24 Dual Cores and 64 GB of RAM, as well as two NVIDIA Titan XP GPUs with 12GB GDDR5X 3840 CUDA cores. The van der Waals (vdW) interactions were cut off at 1.0 nm, and long-range electrostatic interactions were calculated by the Particle Mesh Ewald (PME) [108] with a cut-off of 1.0 nm.

### 4.9. Binding Free Energy Calculations

The free binding energy of hits C1 and C2 as well as the well-known hDHFR inhibitor MTX was calculated by the method MM-PBSA (Molecular Mechanics [MM] with Poisson–Boltzmann Surface Area) [109]. To achieve this goal, we used the software G_mmpbsa [110] in each evaluated enzyme-inhibitor complex. The process to estimate the binding-free energy ∆G_binding_ can be summarized as:

∆G_binding_ = ∆E_MM_ + ∆G_solvation_

E_MM_ = E_bonded_ + E_nonbonded_ = E_bonded_ + (E_vdW_ + E_elec_)

G_solvation_ = G_polar_ + G_nonpolar_

where ∆E_MM_ includes the energy of both bonded and non-bonded interactions according to MM force-field typical parameters, and ∆G_solvation_ represents the sum of electrostatic and non-electrostatic contributions to the solvation-free energy.

### 4.10. Density Functional Theory

Density Functional Theory (DFT) calculations have arisen as a valuable method to provide information regarding the electrostatic characteristics of compounds, and therefore, we used docked conformations of hit compounds C1 and C2 along with active compound MTX as inputs for these calculations. Geometry optimization was carried out using Truhlar’s M062X functional and the basis set 6-31G [111]. The obtained geometry was used for a single-point calculation using the double hybrid B2PLYP-D3 functional and the basis set 6-31G [112]. Afterward, three quantum chemical descriptors, namely, the highest occupied molecular orbital (HOMO), least unoccupied molecular orbital (LUMO), and energy gap (DE) [113] were calculated for all compounds. This provided information about the capacity of the compounds to transfer their energies from a HOMO (electron donor) to a LUMO (electron acceptor) [114].

### 4.11. Chemoinformatics Analysis

We estimated ADME properties of the hit compounds C1 and C2 along with the active compound MTX via the open-access web server SwissADME (http://www.swissadme.ch/; (accessed on 30 October 2022)) [115] directly from the molecular structure of the hit compounds in terms of three categories: pharmacokinetics, drug-likeness, and medicinal chemistry. In the first one, gastrointestinal (GI) absorption, blood–brain barrier (BBB) permeability, and Pgp interaction (known to expel molecules from the brain) were estimated [116], as well as the potential inhibition of three different cytochrome P450 isoforms: CYP1A2, 2C9, and 3A4 [117]. For the second category, we estimated the number of violations for either Lipinski [118], Ghose [119], Veber [120], Egan [121], and Muegge [122] rules as well as the corresponding bioavailability score (likelihood of a compound to exhibit a bioavailability higher than 10% in rat or measurable Caco-2 permeability) [123]. Finally, in the third category, we predicted any PAINS [124] or Brenk [125] structural alerts and estimated a corresponding synthetic accessibility score using a predetermined scale ranging from 1.0 for ‘very easy’ compounds to 10.0 for ‘very difficult’ compounds [115].

### 4.12. Cell Cultures and Experimental Design

Human and mouse melanoma cell lines (A375 and B16, respectively) were grown in Dulbecco’s Modified Eagle’s Medium (DMEM), supplemented with Fetal Bovine Serum (FBS) (10% final concentration). Cells were treated with various concentrations of hit compounds C1 and C2 (Princeton Biomolecular Research, Inc.; purity level >90%) retrieved from the VS process. Methotrexate (MTX) and aminopterin (AMT) (Sigma Aldrich, Inc., Burlington, MA, USA), a pair of well-known human DHFR inhibitors, were included in the study for comparison purposes. All of the compounds were dissolved in DMSO at a final concentration of 0.2% (v/v) before addition to the cells in microtiter wells. Additionally, cell viability was determined at the highest drug concentration tested (11 µg/mL) for all compounds at different times (12 h, 24 h, 36 h, and 48 h) post-treatment (each sample was analyzed in triplicate). We visualized the cells not exposed (control group) and the cells exposed to compounds C1, C2, MTX, or AMT using an inverted microscope (Nikon Microscope Eclipse Ti, Tokyo, Japan) and then we photomicrographed all experimental groups (Nikon Digital Sight DS U3, Tokyo, Japan).

### 4.13. Determination of Cell Viability by MTT and Trypan Blue Assays

Cell viability was determined by MTT (3-[4,5-dimethylthiazol-2-yl]-2,5 diphenyl tetrazolium bromide) reduction assay (Sigma Aldrich, Inc.) [126,127]. Calibration curves were constructed for each cell line (A375 and B16) using a serial two-fold dilution (from a known density) of melanoma cells. We estimated the amount of MTT formazan product in terms of absorbance at a wavelength of 570 nm and a reference wavelength of 750 nm. In addition, we performed a Trypan Blue (TB) assay [128,129] and estimated the number of viable cells using a hemocytometer. Concentrations and time intervals in this procedure were equivalent to those used in MTT assays.

### 4.14. Statistical Analysis

Results were expressed as mean ± SD with n referring to the number of experiments. Linear regression analysis was carried out using the Origin software (Micrococcal Software Inc., One Roundhouse Plaza, Northampton, MA, USA). We determined differences between means using the Student’s *t*-test. Lethal concentration 50 (LC50) values, which represent the concentrations of the test agent, caused a 50% reduction in cell number compared to the control group. A value of *p* < 0.05 was considered significant.

## 5. Conclusions

Although progress has been made in the understanding of melanoma, the discovery of new drugs remains an urgent need largely because of the steadily increasing incidence rate and their resistance to antifolates and many other chemotherapeutic drugs. In this context, the ‘non-classical’ DHFR inhibitors provide a valuable opportunity to cope with several melanoma-specific resistance mechanisms that made them insensitive to the classical inhibitors of this enzyme and others involved in the metabolic pathway of the folate cycle. The methodological strategy adopted in this study provided a starting point for the development of future inhibitors because of their unique characteristics: the hit compounds identified after the in silico protocol were predicted as diverse, potent, and developable and, among them, C1 and C2 proved active against melanoma growth in vitro. Since the inherent flexibility of pharmacophore models to use 3D chemical descriptors facilitated the identification of compounds with a lipophilic side chain (instead of glutamate), it seems likely that this methodology might be used for other enzymes in cases where a particular fragment requires to be replaced. We expect that our findings will assist in the discovery and development of future drugs displaying activity against this deadly form of skin cancer through the blocking of protein targets implicated in cell growth and proliferation. However, we are not unaware that C1, C2, or other hits may also inhibit other enzymes in the folate cycle, or even be repurposed to treat other tumors or opportunistic infections—as shown for other antifolates—and, thus, further studies will be necessary to develop soon to help further elucidate their mechanism of action.

## Figures and Tables

**Table 1:** Chemoinformatics analysis for hit dataset, C1, and C2 after the virtual screening. Molecular weight (MW), lipophilicity (logP), number of rings, and number of aromatic rings were listed for the entire hit dataset as well as compounds C1 (ZINC00907702) and C2 (ZINC20102709). Molecular weight values were calculated in Daltons (Da). ^a^ All the physicochemical properties for the hit dataset were estimated as average values.

Physicochemical Property	Hit Dataset ^a^	C1	C2
MW (Da)	3.766	351.43	452.47
LogP	3.544	4.260	4.800
Number of rings	3.421	3	6
Number of aromatic rings	2.537	3	2

## Data Availability

The datasets used and/or analyzed during the current study are available from the corresponding author upon reasonable request.

## Supplementary Materials

The following supporting information can be downloaded at: https://www.mdpi.com/article/10.3390/ijms232213946/s1, Figure S1. ROC curves of pharmacophore models generated in Ligand Scout. The performance of the pharmacophore models (A) Pharm-A and (B) Pharm-B was estimated by ROC curve analysis using well-known active and inactive compounds in a decoy set. The red and blue curves are the ROC curves for Pharm-A and Pharm-B, respectively. Accuracy was calculated according to the following formula: (TP+TN)/(TP+FP+TN+FN) [40]; Figure S2. Structural alignment of hDHFR crystals selected for the conformational ensemble. A) Full and zoomed view of the three selected 3D structures (PDB codes:1KMV [36], 1U72 [37] and 3NXV [38]), emphasizing in the clustering of their Phe31 side-chains (stick representation). Dunbrack, Dynameomics and Richardson rotamers with highest probability (wire representation) are illustrated in gold, cyan and lime green, respectively. B) Janin plot describing the Phe31 dihedral angles Chi1 and Chi2. The combinations of these angles in the selected structures are illustrated as dark red points; Figure S3. Hierarchical clustering and multidimensional scaling on hit dataset after virtual screening. Average hierarchical clustering (A) and multidimensional scaling (B) generated in ChemMine Tools based on pairwise ligand similarity for 173 ligands. The polar tree layout representing the ligands in our dataset was generated using the graphical software FigTree, and individual hit compounds at the end of each branch are labeled and colored by lipophilicity (logP; internal colored circle) and by molecular weight (MW; external colored circle). Hit compounds C1 and C2 are shown as shaded lines in (A) and as red-encircled spheres in (B); Figure S4. Chemical structures of hit compounds C1 and C2. The 2D chemical structures of compounds C1 (ZINC00907702; 2-(4,6-diaminopyrimidine-1,3-diium-2-yl)sulfanyl-*N*,*N*-diphenylacetamide) and C2 (ZINC20102709; *N*-[3-[[2-(4,6-diaminopyrimidin-2-yl)sulfanylacetyl]amino]phenyl]adamantane-1-carboxamide), identified after virtual screening procedures, were both prepared with MarvinSketch v17.14, 2017, ChemAxon; Figure S5. Overlay of pharmacophore model Pharm-B with hits C1 and C2. These hit compounds are showed aligned with PharmB model previously used in the screening in both 3D (shown here as (A) for C1 and (C) for C2) and 2D representations (shown here as (B) for C1 and (D) for C2), respectively. Hydrogen bond donor (HBD), hydrophobic group (HY), and aromatic ring (AR) features are colored green, gold and blue, respectively. Excluded volume spheres are illustrated in gray color; Figure S6. Docking pose analysis for hit compounds C1 and C2 including (and not including) water molecules. Superimposed 2D docked poses of MTX (A), hit compounds C1 (B) and C2 (C), and a selected DHFR decoy (D) generated by the program Ligplot+. H-bonds are shown as green dashed lines with their corresponding distances in angstroms (Å) and hydrophobic are indicated by spoked arcs pointing towards each compound. Compounds C1 and C2 were predicted to interact with a conserved water molecule in human DHFR active site (inside of dashed line green circle), as observed also for MTX but not for the hDHFR decoy. A structurally conserved water molecule was predicted to facilitate the interaction with hDHFR active site residues Glu30 and Trp24; Figure S7. Docking pose analysis for hit compounds C1 and C2 by FlexX. 2D docked diagrams generated by the program FlexX and visualized by PoseView for either C1 (A) and C2 (B) bound into hDHFR active site. Both compounds were predicted to interact with a conserved water molecule in human DHFR active site (inside of a dashed line red circle in each case). H-bonds are indicated here as dashed lines, and hydrophobic interactions are shown as green solid lines; Figure S8. RMSD plot of the backbone atoms of hDHFR. The RMSD calculation was performed on the Cα atoms of hDHFR over 250 ns of MD simulations for the (a) unbound form, and the bound form complexed with (b) MTX, (c) C1 and (d) C2. The gray boxes in each graph represent equilibration time and, hence, this period was not considered for further analysis; Figure S9. Potential energy plot obtained from the MD simulation for hDHFR. Calculation of potential energy fluctuation curves was performed on the atoms of enzymes hDHFR over 250 ns of MD simulations for the unbound form (A) and the bound form complexed with active compound MTX (B) and the hit compounds C1 (C) and C2 (D); Figure S10. Kinetic energy plot obtained from the MD simulation for hDHFR. Calculation of kinetic energy fluctuation curves was performed on the atoms of enzymes hDHFR over 250 ns of MD simulations for the unbound form (A) and the bound form complexed with active compound MTX (B) and the hit compounds C1 (C) and C2 (D); Figure S11. Total energy plot obtained from the MD simulation for hDHFR. Calculation of total energy fluctuation curves was performed on the atoms of enzymes hDHFR over 250 ns of MD simulations for the unbound form (A) and the bound form complexed with active compound MTX (B) and the hit compounds C1 (C) and C2 (D); Figure S12. Volume plot obtained from the MD simulation for hDHFR. Calculation of volume fluctuation curves was performed on the atoms of enzymes hDHFR over 250 ns of MD simulations for the unbound form (A) and the bound form complexed with active compound MTX (B) and the hit compounds C1 (C) and C2 (D); Figure S13. Temperature plot obtained from the MD simulation for hDHFR. Calculation of temperature fluctuation curves was performed on the atoms of enzymes hDHFR over 250 ns of MD simulations for the unbound form (A) and the bound form complexed with active compound MTX (B) and the hit compounds C1 (C) and C2 (D); Figure S14. Pressure plot obtained from the MD simulation for hDHFR. Calculation of pressure fluctuation curves was performed on the atoms of enzymes hDHFR over 250 ns of MD simulations for the unbound form (A) and the bound form complexed with active compound MTX (B) and the hit compounds C1 (C) and C2 (D); Figure S15. Density plot obtained from the MD simulation for hDHFR. Calculation of density fluctuation curves was performed on the atoms of enzymes hDHFR over 250 ns of MD simulations for the unbound form (A) and the bound form complexed with active compound MTX (B) and the hit compounds C1 (C) and C2 (D); Figure S16. Binding energy plot for hDHFR. Binding energy calculation was carried out for hDHFR over 250 ns of MD simulations in complex with reference compound MTX (A), C1 (B), and C2 (C); Figure S17. Contribution of each residue of hDHFR to ligand binding. Contribution from each residue was estimated for hDHFR over 250 ns of MD simulations complexed with MTX (A), C1 (B) and C2 (C). The horizontal discontinuous lines in each graph represent an energy value equal to −3 kJ/mol; Figure S18. Calibration curves for dose-dependent assays. Absorbance versus cell number for human (A375; left panel), and mouse (B16; right panel) melanoma cell lines is plotted. The number of cells is directly proportional to the level of the formazan product generated (R2 = 0.9998 and R2 = 0.9995 for A375 and B16 melanoma cells, respectively) after quantification at 570nm; Figure S19. Time-dependent effect of compounds C1 and C2 on human melanoma cell line A735. Cell viability determined by MTT reductase activity after exposure to hit compounds C1 (gray-colored line) C2 (black-colored line) during a period of 12, 24 36 and 48 h, respectively. The concentration used for each compound was 11 µg/mL; Figure S20. Effect on A375 cells after exposure to C1 and C2 compared to MTX and AMT. Human (A375) melanoma cells were seeded into a 48-well plate overnight and cell viability was determined by MTT reductase activity and trypan blue (TB) exclusion assay after exposure to hit compounds C1 and C2, as well as reference compounds MTX and AMT, at a concentration of 11 µg/mL during a period of 48 h. A moderate reduction on cell viability was estimated for human melanoma cells after exposure to hit compound C1, but to a lesser extent compared to that observed for MTX and AMT. Conversely, a remarkable reduction on cell viability after exposure to hit compound C2 was estimated, which was higher than that observed for reference compounds (see text for details); Table S1. Analysis of critical amino acid residues for hDHFR inhibition. A total of 54 native co-crystal structures in Protein Data Bank was analyzed in order to determine the amino acids interacting with different DHFR inhibitors. Any marked amino acid is an indication of its role in protein-ligand interaction in the corresponding PDB structure; Table S2. Statistical parameters from screening compounds in decoy set. a Area under ROC curve: ∑_(x = 2)^N〖TPR(x)[FPR(x) − FPR(x − 1)]〗 [130]; b Enrichment factor: [(TP*D)/((TP + FP)*A))] [131]; c Sensitivity, also called Recall or True Positive rate (TPR): [TP/(TP + FN)] [130]; d Specificity, also called True Negative Rate (TNR): [TN/(TN + FP)] [130]; Table S3. Predicted binding energy and Ki among hit compounds C1, C2, MTX and a hDHFR decoy. Binding energy was measured in kcal/mol and inhibition constant (Ki) values were calculated in nanomolar (nM) scale; Table S4. Results of MM/PBSA and electronic parameters calculated for active compound MTX and hit compounds C1 and C2. Binding energy was measured in kJ/mol and HOMO, LUMO and energy gap values were all estimated in electron volts (eV); Table S5. ADME properties of the active compound MTX and the hit compounds C1 and C2 identified after the VS procedures. The different properties associated with pharmacokinetics, drug-likeness, and medicinal chemistry were estimated using the web-server SwissADME [115]; Table S6. Top 10 highest resolution hDHFR crystal structures. Every hDHFR 3D structure is reported in terms of its corresponding 4-character unique PDB ID, resolution (Å) and its co-crystallized ligand.

## References

[1] Bhagat K., Kumar N., Kaur Gulati H., Sharma A., Kaur A., Singh J.V., Singh H., Bedi P.M.S. (2022). Dihydrofolate Reductase Inhibitors: Patent Landscape and Phases of Clinical Development (2001–2021). Expert Opin. Ther. Pat..

[2] Raimondi M.V., Randazzo O., La Franca M., Barone G., Vignoni E., Rossi D., Collina S. (2019). DHFR Inhibitors: Reading the Past for Discovering Novel Anticancer Agents. Molecules.

[3] Wan Q., Bennett B.C., Wymore T., Li Z., Wilson M.A., Brooks C.L., Langan P., Kovalevsky A., Dealwis C.G. (2021). Capturing the Catalytic Proton of Dihydrofolate Reductase: Implications for General Acid—Base Catalysis. ACS Catal..

[4] Tuttle L.M., Dyson H.J., Wright P.E. (2014). Side Chain Conformational Averaging in Human Dihydrofolate Reductase. Biochemistry.

[5] Ko A., Minev B.R. (2011). Folate Antagonists. Cancer Management in Man: Chemotheraphy, Biological Therapy, Hyperthermia and Supporting Measures (Cancer Growth and Progression).

[6] Laanpere M., Altmäe S., Stavreus-Evers A., Nilsson T.K., Yngve A., Salumets A. (2010). Folate-Mediated One-Carbon Metabolism and Its Effect on Female Fertility and Pregnancy Viability. Nutr. Rev..

[7] Willson J. (2022). Structural Study Could Aid Design of Antifolates. Nat. Rev. Cancer.

[8] Walling J. (2006). From Methotrexate to Pemetrexed and beyond. A Review of the Pharmacodynamic and Clinical Properties of Antifolates. Invest. New Drugs.

[9] Hamed K.M., Dighriri I.M., Baomar A.F., Alharthy B.T., Alenazi F.E., Alali G.H., Alenazy R.H., Alhumaidi N.T., Alhulayfi D.H., Alotaibi Y.B. (2022). Overview of Methotrexate Toxicity: A Comprehensive Literature Review. Cureus.

[10] Uchihara Y., Komori R., Tago K., Tamura H., Funakoshi-Tago M. (2019). Methotrexate Significantly Induces Apoptosis by Inhibiting STAT3 Activation in NPM-ALK-Positive ALCL Cells. Biochem. Pharmacol..

[11] Salem I.M., Mostafa S.M., Salama I., El-Sabbagh O.I., A H Hegazy W., Ibrahim T.S. (2022). Human Dihydrofolate Reductase Inhibition Effect of 1-Phenylpyrazolo[3,4-d]Pyrimidines: Synthesis, Antitumor Evaluation and Molecular Modeling Study. Bioorg. Chem..

[12] Rodríguez-López J.N., Sanchez-del-Campo L., Saez-Ayala M., Montenegro M.F., Cabezas-Herrera J., Murph M. (2011). Novel Antifolates as Prodrugs for the Treatment of Melanoma. Research on Melanoma—A Glimpse into Current Directions and Future Trends.

[13] Sánchez-del-Campo L., Montenegro M.F., Cabezas-Herrera J., Rodríguez-López J.N. (2009). The Critical Role of Alpha-Folate Receptor in the Resistance of Melanoma to Methotrexate. Pigment Cell Melanoma Res..

[14] Wang H., Tran T.T., Duong K.T., Nguyen T., Le U.M. (2022). Options of Therapeutics and Novel Delivery Systems of Drugs for the Treatment of Melanoma. Mol. Pharm..

[15] Matthews N.H., Wen-Qing L., Qureshi A.A., Weinstock M.A., Cho E., Ward W., Farma J. (2017). Epidemiology of Melanoma. Cutaneous Melanoma: Etiology and Therapy.

[16] Kosary C.L., Altekruse S.F., Ruhl J., Lee R., Dickie L. (2014). Clinical and Prognostic Factors for Melanoma of the Skin Using SEER Registries: Collaborative Stage Data Collection System, Version 1 and Version 2. Cancer.

[17] Wróbel A., Arciszewska K., Maliszewski D., Drozdowska D. (2020). Trimethoprim and Other Nonclassical Antifolates an Excellent Template for Searching Modifications of Dihydrofolate Reductase Enzyme Inhibitors. J. Antibiot. (Tokyo).

[18] El-Subbagh H.I., Hassan G.S., El-Messery S.M., Al-Rashood S.T., Al-Omary F.A.M., Abulfadl Y.S., Shabayek M.I. (2014). Nonclassical Antifolates, Part 5. Benzodiazepine Analogs as a New Class of DHFR Inhibitors: Synthesis, Antitumor Testing and Molecular Modeling Study. Eur. J. Med. Chem..

[19] Dewar S., Sienkiewicz N., Ong H.B., Wall R.J., Horn D., Fairlamb X.A.H. (2016). The Role of Folate Transport in Antifolate Drug Action in Trypanosoma Brucei. J. Biol. Chem..

[20] Wang Z., Sun H., Shen C., Hu X., Gao J., Li D., Cao D., Hou T. (2020). Combined Strategies in Structure-Based Virtual Physical Chemistry Chemical Physics Accepted Manuscript. Phys. Chem. Chem. Phys..

[21] Slater O., Kontoyianni M., Slater O., Kontoyianni M. (2019). The Compromise of Virtual Screening and Its Impact on Drug Discovery The Compromise of Virtual Screening and Its Impact on Drug Discovery. Expert Opin. Drug Discov..

[22] da Silva Rocha S.F.L., Olanda C.G., Fokoue H.H., Sant’Anna C.M.R. (2019). Virtual Screening Techniques in Drug Discovery: Review and Recent Applications. Curr. Top. Med. Chem..

[23] Biswas R., Chowdhury N., Mukherjee R., Bagchi A. (2018). Identification and Analyses of Natural Compounds as Potential Inhibitors of TRAF6-Basigin Interactions in Melanoma Using Structure-Based Virtual Screening and Molecular Dynamics Simulations. J. Mol. Graph. Model..

[24] Giordano D., Biancaniello C., Argenio M.A., Facchiano A. (2022). Drug Design by Pharmacophore and Virtual Screening Approach. Pharmaceuticals.

[25] Sanders M.P.A., McGuire R., Roumen L., de Esch I.J.P., de Vlieg J., Klomp J.P.G., de Graaf C. (2012). From the Protein’s Perspective: The Benefits and Challenges of Protein Structure-Based Pharmacophore Modeling. Med. Chem. Commun..

[26] Wallach I. (2011). Pharmacophore Inference and Its Application to Computational Drug Discovery. Drug Dev. Res..

[27] Ballante F., Kooistra A.J., Kampen S., De Graaf C., Carlsson J. (2022). Structure-Based Virtual Screening for Ligands of G Protein-Coupled Receptors: What Can Molecular Docking Do for You?. Pharmacol. Rev..

[28] Hosseini M., Chen W., Xiao D., Wang C. (2021). Computational Molecular Docking and Virtual Screening Revealed Promising SARS-CoV-2 Drugs. Precis. Clin. Med..

[29] Stanzione F., Giangreco I., Cole J.C. (2021). Use of Molecular Docking Computational Tools in Drug Discovery. Progress in Medicinal Chemistry.

[30] Hernández-Rodríguez M., Rosales-Hernández M.C., Mendieta-Wejebe J.E., Martínez-Archundia M., Basurto J.C. (2016). Current Tools and Methods in Molecular Dynamics (MD) Simulations for Drug Design. Curr. Med. Chem..

[31] Onyango H., Odhiambo P., Angwenyi D., Okoth P. (2022). In Silico Identification of New Anti-SARS-CoV-2 Main Protease (M(pro)) Molecules with Pharmacokinetic Properties from Natural Sources Using Molecular Dynamics (MD) Simulations and Hierarchical Virtual Screening. J. Trop. Med..

[32] Al-Shar’i N., Musleh S.S. (2022). CHK1 Kinase Inhibition: Identification of Allosteric Hits Using MD Simulations, Pharmacophore Modeling, Docking and MM-PBSA Calculations. Mol. Divers..

[33] Starosyla S.A., Volynets G.P., Protopopov M.V., Bdzhola V.G., Pashevin D.O., Polishchuk V.O., Kozak T.O., Stroi D.O., Dosenko V.E., Yarmoluk S.M. (2022). Pharmacophore Modeling, Docking and Molecular Dynamics Simulation for Identification of Novel Human Protein Kinase C Beta (PKCβ) Inhibitors. Struct. Chem..

[34] Thangavel N., Albratty M. (2022). Pharmacophore Model-Aided Virtual Screening Combined with Comparative Molecular Docking and Molecular Dynamics for Identification of Marine Natural Products as SARS-CoV-2 Papain-like Protease Inhibitors. Arab. J. Chem..

[35] Dhameliya T.M., Nagar P.R., Gajjar N.D. (2022). Systematic Virtual Screening in Search of SARS CoV-2 Inhibitors against Spike Glycoprotein: Pharmacophore Screening, Molecular Docking, ADMET Analysis and MD Simulations. Mol. Divers..

[36] Klon A.E., Héroux A., Ross L.J., Pathak V., Johnson C.A., Piper J.R., Borhani D.W. (2002). Atomic Structures of Human Dihydrofolate Reductase Complexed with NADPH and Two Lipophilic Antifolates at 1.09 Å and 1.05 Å Resolution. J. Mol. Biol..

[37] Cody V., Luft J.R., Pangborn W. (2005). Understanding the Role of Leu22 Variants in Methotrexate Resistance: Comparison of Wild-Type and Leu22Arg Variant Mouse and Human Dihydrofolate Reductase Ternary Crystal Complexes with Methotrexate and NADPH. Acta Crystallogr. D Biol. Crystallogr..

[38] Cody V., Piraino J., Pace J., Li W., Gangjee A. (2010). Preferential Selection of Isomer Binding from Chiral Mixtures: Alternate Binding Modes Observed for the E and Z Isomers of a Series of 5-Substituted 2,4-Diaminofuro[2,3-d]Pyrimidines as Ternary Complexes with NADPH and Human Dihydrofolate Reductase. Acta Crystallogr. Sect. D Biol. Crystallogr..

[39] Penhallurick R.W., Durnal M.D., Harold A., Ichiye T. (2021). Adaptations for Pressure and Temperature in Dihydrofolate Reductases. Microorganisms.

[40] Rizzi A., Fioni A. (2008). Virtual Screening Using PLS Discriminant Analysis and ROC Curve Approach: An Application Study on PDE4 Inhibitors. J. Chem. Inf. Model..

[41] Al-Nadaf A.H., Taha M.O. (2011). Discovery of New Renin Inhibitory Leads via Sequential Pharmacophore Modeling, QSAR Analysis, in Silico Screening and in Vitro Evaluation. J. Mol. Graph. Model..

[42] Kim H.J., Choo H., Cho Y.S., No K.T., Pae A.N. (2008). Novel GSK-3β Inhibitors from Sequential Virtual Screening. Bioorg. Med. Chem..

[43] Bowman A.L., Lerner M.G., Carlson H.A. (2007). Protein Flexibility and Species Specificity in Structure-Based Drug Discovery: Dihydrofolate Reductase as a Test System. J. Am. Chem. Soc..

[44] Sawaya M.R., Kraut J. (1997). Loop and Subdomain Movements in the Mechanism of Escherichia Coli Dihydrofolate Reductase: Crystallographic Evidence. Biochemistry.

[45] Shrimpton P., Mullaney A., Allemann R.K. (2003). Functional Role for Tyr 31 in the Catalytic Cycle of Chicken Dihydrofolate Reductase. Proteins Struct. Funct. Genet..

[46] Anderson A.C., Wright D.L. (2005). The Design and Docking of Virtual Compound Libraries to Structures of Drug Targets. Curr. Comput.—Aided Drug Des..

[47] Ritchie T.J., Macdonald S.J.F. (2009). The Impact of Aromatic Ring Count on Compound Developability—Are Too Many Aromatic Rings a Liability in Drug Design?. Drug Discov. Today.

[48] Scior T., Bender A., Tresadern G., Medina-Franco J.L., Martínez-Mayorga K., Langer T., Cuanalo-Contreras K., Agrafiotis D.K. (2012). Recognizing Pitfalls in Virtual Screening: A Critical Review. J. Chem. Inf. Model..

[49] Zhang X., Zhou X., Kisliuk R.L., Piraino J., Cody V., Gangjee A. (2011). Design, Synthesis, Biological Evaluation and X-Ray Crystal Structure of Novel Classical 6,5,6-Tricyclic Benzo[4,5]Thieno[2,3-d]Pyrimidines as Dual Thymidylate Synthase and Dihydrofolate Reductase Inhibitors. Bioorg. Med. Chem..

[50] Göksel Y., Zor K., Rindzevicius T., Thorhauge Als-Nielsen B.E., Schmiegelow K., Boisen A. (2021). Quantification of Methotrexate in Human Serum Using Surface-Enhanced Raman Scattering—Toward Therapeutic Drug Monitoring. ACS Sens..

[51] Panecka-Hofman J., Pöhner I., Spyrakis F., Zeppelin T., Di Pisa F., Dello Iacono L., Bonucci A., Quotadamo A., Venturelli A., Mangani S. (2017). Comparative Mapping of On-Targets and off-Targets for the Discovery of Anti-Trypanosomatid Folate Pathway Inhibitors. Biochim. Biophys. Acta—Gen. Subj..

[52] Kitchen D.B., Decornez H., Furr J.R., Bajorath J. (2004). Docking and Scoring in Virtual Screening for Drug Discovery: Methods and Applications. Nat. Rev. Drug Discov..

[53] Ripphausen P., Nisius B., Peltason L., Bajorath J. (2010). Quo Vadis, Virtual Screening? A Comprehensive Survey of Prospective Applications. J. Med. Chem..

[54] Miar M., Shiroudi A., Pourshamsian K., Oliaey A.R., Hatamjafari F. (2021). Theoretical Investigations on the HOMO–LUMO Gap and Global Reactivity Descriptor Studies, Natural Bond Orbital, and Nucleus-Independent Chemical Shifts Analyses of 3-Phenylbenzo[d]Thiazole-2(3H)-Imine and Its Para-Substituted Derivatives: Solvent and Subs. J. Chem. Res..

[55] Pegu D., Deb J., Van Alsenoy C., Sarkar U. (2017). Theoretical Investigation of Electronic, Vibrational, and Nonlinear Optical Properties of 4-Fluoro-4-Hydroxybenzophenone. Spectrosc. Lett..

[56] Wróbel A., Baradyn M., Ratkiewicz A., Drozdowska D. (2021). Synthesis, Biological Activity, and Molecular Dynamics Study of Novel Series of a Trimethoprim Analogs as Multi-targeted Compounds: Dihydrofolate Reductase (Dhfr) Inhibitors and Dna-binding Agents. Int. J. Mol. Sci..

[57] Amusengeri A., Tata R.B., Bishop Ö.T. (2020). Understanding the Pyrimethamine Drug Resistance Mechanism via Combined Molecular Dynamics and Dynamic Residue Network Analysis. Molecules.

[58] Jovanović M., Gruden-Pavlović M., Zlatović M. (2015). Stabilizing Non-Covalent Interactions of Ligand Aromatic Moieties and Proline in Ligand-Protein Systems. Monatshefte fur Chemie.

[59] Zondlo N.J. (2013). Aromatic-Proline Interactions: Electronically Tunable CH/π Interactions. Acc. Chem. Res..

[60] Biedermannova L., E (2008). Riley, K.; Berka, K.; Hobza, P.; Vondrasek, J. Another Role of Proline: Stabilization Interactions in Proteins and Protein Complexes Concerning Proline and Tryptophane. Phys. Chem. Chem. Phys..

[61] Leitgeb B., Tóth G. (2005). Aromatic-Aromatic and Proline-Aromatic Interactions in Endomorphin-1 and Endomorphin-2. Eur. J. Med. Chem..

[62] Baghel P., Roy A., Verma S., Satapathy T., Bahadur S. (2020). Amelioration of Lipophilic Compounds in Regards to Bioavailability as Self-Emulsifying Drug Delivery System (SEDDS). Futur. J. Pharm. Sci..

[63] Psimadas D., Georgoulias P., Valotassiou V., Loudos G. (2012). Effect of Lipophilicity on the Bioavailability of Drugs After Percutaneous Administration by Dissolving Microneedles. J. Pharm. Sci..

[64] Orzeszko B., Kazimierczuk Z., Maurin J.K., Laudy A.E., Starościak B.J., Vilpo J., Vilpo L., Balzarini J., Orzeszko A. (2004). Novel Adamantylated Pyrimidines and Their Preliminary Biological Evaluations. Farm. (Società Chim. Ital. 1989).

[65] Zahid M., Yasin K.A., Akhtar T., Rama N.H., Hameed S., Al-Masoudi N.A., Loddo R., La Colla P. (2009). Synthesis and in Vitro Antiproliferative Activity of New Adamantylthiazolyl-1,3,4-Oxadiazoles. Ark. Online J. Org. Chem..

[66] Gangjee A., Jain H., Kurup S. (2007). Recent Advances in Classical and Non-Classical Antifolates as Antitumor and Antiopportunistic Infection Agents: Part I. Anticancer Agents Med. Chem..

[67] Gangjee A., Jain H., Kurup S. (2008). Recent Advances in Classical and Non-Classical Antifolates as Antitumor and Antiopportunistic Infection Agents: Part II. Anticancer Agents Med. Chem..

[68] Scocchera E., Reeve S.M., Keshipeddy S., Lombardo M.N., Hajian B., Sochia A.E., Alverson J.B., Priestley N.D., Anderson A.C., Wright D.L. (2016). Charged Nonclassical Antifolates with Activity Against Gram-Positive and Gram-Negative Pathogens. ACS Med. Chem. Lett..

[69] Bhatt J.D., Chudasama C.J., Patel K.D. (2017). Diarylpyrazole Ligated Dihydropyrimidine Hybrids as Potent Non-Classical Antifolates and Their Efficacy Against Plasmodium Falciparum. Arch. Pharm. (Weinheim).

[70] Liu H., Qin Y., Zhai D., Zhang Q., Gu J., Tang Y., Yang J., Li K., Yang L., Chen S. (2019). Antimalarial Drug Pyrimethamine Plays a Dual Role in Antitumor Proliferation and Metastasis through Targeting DHFR and TP. Mol. Cancer Ther..

[71] McGuire J.J. (2003). Anticancer Antifolates: Current Status and Future Directions. Curr. Pharm. Des..

[72] Wolber G., Langer T. (2005). LigandScout: 3-D Pharmacophores Derived from Protein-Bound Ligands and Their Use as Virtual Screening Filters. J. Chem. Inf. Model..

[73] Pettersen E.F., Goddard T.D., Huang C.C., Couch G.S., Greenblatt D.M., Meng E.C., Ferrin T.E. (2004). UCSF Chimera--a Visualization System for Exploratory Research and Analysis. J. Comput. Chem..

[74] Mills J.E.J., Dean P.M. (1996). Three-Dimensional Hydrogen-Bond Geometry and Probability Information from a Crystal Survey. J. Comput. Aided. Mol. Des..

[75] Tsai J., Taylor R., Chothia C., Gerstein M. (1999). The Packing Density in Proteins: Standard Radii and Volumes. J. Mol. Biol..

[76] Irwin J.J., Shoichet B.K. (2005). ZINC—A Free Database of Commercially Available Compounds for Virtual Screening. J. Chem. Inf. Model..

[77] Irwin J.J., Sterling T., Mysinger M.M., Bolstad E.S., Coleman R.G. (2012). ZINC: A Free Tool to Discover Chemistry for Biology. J. Chem. Inf. Model..

[78] Sterling T., Irwin J.J. (2015). ZINC 15—Ligand Discovery for Everyone. J. Chem. Inf. Model..

[79] Huang N., Shoichet B.K., Irwin J.J. (2006). Benchmarking Sets for Molecular Docking. J. Med. Chem..

[80] Kokh D.B., Wade R.C., Wenzel W. (2011). Receptor Flexibility in Small-Molecule Docking Calculations. Wiley Interdiscip. Rev. Comput. Mol. Sci..

[81] Dunbrack R.L. (2002). Rotamer Libraries in the 21st Century. Curr. Opin. Struct. Biol..

[82] Scouras A.D., Daggett V. (2011). The Dynameomics Rotamer Library: Amino Acid Side Chain Conformations and Dynamics from Comprehensive Molecular Dynamics Simulations in Water. Protein Sci..

[83] Lovell S.C., Word J.M., Richardson J.S., Richardson D.C. (2000). The Penultimate Rotamer Library. Proteins Struct. Funct. Bioinform..

[84] Janin J., Wodak S., Levitt M., Maigret B. (1978). Conformation of Amino Acid Side-Chains in Proteins. J. Mol. Biol..

[85] Craig I.R., Essex J.W., Spiegel K. (2010). Ensemble Docking into Multiple Crystallographically Derived Protein Structures: An Evaluation Based on the Statistical Analysis of Enrichments. J. Chem. Inf. Model..

[86] Bottegoni G., Kufareva I., Totrov M., Abagyan R. (2009). Four-Dimensional Docking: A Fast and Accurate Account of Discrete Receptor Flexibility in Ligand Docking. J. Med. Chem..

[87] Morris G.M., Goodsell D.S., Halliday R.S., Huey R., Hart W.E., Belew R.K., Olson A.J. (1998). Automated Docking Using a Lamarckian Genetic Algorithm and an Empirical Binding Free Energy Function. J. Comput. Chem..

[88] Malinauskas T. ADL High-Throughput Molecular Docking Using Free Tools ZINC 8, AutoDockTools 1.5.2 and Docker 1.0. https://web.archive.org/web/20090410151007/http://users.ox.ac.uk/~jesu1458/docker/.

[89] Morris G.M., Huey R., Lindstrom W., Sanner M.F., Belew R.K., Goodsell D.S., Olson A.J. (2009). AutoDock4 and AutoDockTools4: Automated Docking with Selective Receptor Flexibility. J. Comput. Chem..

[90] Backman T.W.H., Cao Y., Girke T. (2011). ChemMine Tools: An Online Service for Analyzing and Clustering Small Molecules. Nucleic Acids Res..

[91] Rambaut A. FigTree v1.4.3. http://tree.bio.ed.ac.uk/software/figtree/.

[92] Willett P. (2006). Similarity-Based Virtual Screening Using 2D Fingerprints. Drug Discov. Today.

[93] Sander T. Openmolecules.Org. http://www.openmolecules.org/propertyexplorer/applet.html.

[94] Kumar A., Zhang K.Y.J. (2013). Investigation on the Effect of Key Water Molecules on Docking Performance in CSARdock Exercise. J. Chem. Inf. Model..

[95] Thilagavathi R., Mancera R.L. (2010). Ligand-Protein Cross-Docking with Water Molecules. J. Chem. Inf. Model..

[96] Hendlich M., Bergner A., Günther J., Klebe G. (2003). Relibase: Design and Development of a Database for Comprehensive Analysis of Protein-Ligand Interactions. J. Mol. Biol..

[97] Maier J.A., Martinez C., Kasavajhala K., Wickstrom L., Hauser K.E., Simmerling C. (2015). Ff14SB: Improving the Accuracy of Protein Side Chain and Backbone Parameters from Ff99SB. J. Chem. Theory Comput..

[98] Wang J., Wolf R.M., Caldwell J.W., Kollman P.A., Case D.A. (2004). Development and Testing of a General Amber Force Field. J. Comput. Chem..

[99] Frisch M.J., Trucks G.W., Schlegel H.B., Scuseria G.E., Robb M.A., Cheeseman J.R., Scalmani G., Barone V., Petersson G.A., Nakatsuji H. (2016). Gaussian 16 Rev. C.01 2016.

[100] Abraham M.J., Murtola T., Schulz R., Páll S., Smith J.C., Hess B., Lindah E. (2015). Gromacs: High Performance Molecular Simulations through Multi-Level Parallelism from Laptops to Supercomputers. SoftwareX.

[101] Páll S., Abraham M.J., Kutzner C., Berk H., Erik L. (2015). Tackling Exascale Software Challenges in Molecular Dynamics Simulations with GROMACS. Solving Software Challenges for Exascale.

[102] Hess B., Kutzner C., Van Der Spoel D., Lindahl E. (2008). GROMACS 4: Algorithms for Highly Efficient, Load-Balanced, and Scalable Molecular Simulation. J. Chem. Theory Comput..

[103] Jorgensen W.L., Chandrasekhar J., Madura J.D., Impey R.W., Klein M.L. (1983). Comparison of Simple Potential Functions for Simulating Liquid Water. J. Chem. Phys..

[104] Meza J.C. (2010). Steepest Descent. Wiley Interdiscip. Rev. Comput. Stat..

[105] Bussi G., Donadio D., Parrinello M. (2007). Canonical Sampling through Velocity Rescaling. J. Chem. Phys..

[106] Fias S., Van Damme S., Bultinck P. (2008). Multidimensionality of Delocalization Indices and Nucleus Independent Chemical Shifts in Polycyclic Aromatic Hydrocarbons. J. Comput. Chem..

[107] Hess B., Bekker H., Berendsen H.J.C., Fraaije J.G.E.M. (1997). LINCS: A Linear Constraint Solver for Molecular Simulations. J. Comput. Chem..

[108] Darden T., York D., Pedersen L. (1993). Particle Mesh Ewald: An N·log(N) Method for Ewald Sums in Large Systems. J. Chem. Phys..

[109] Genheden S., Ryde U. (2015). The MM/PBSA and MM/GBSA Methods to Estimate Ligand-Binding Affinities. Expert Opin. Drug Discov..

[110] Kumari R., Kumar R., Source O., Discovery D., Lynn A. (2014). G_mmpbsa—A GROMACS Tool for High-Throughput MM-PBSA Calculations. J. Chem. Inf. Model..

[111] Zhao Y., Truhlar D.G. (2008). The M06 Suite of Density Functionals for Main Group Thermochemistry, Thermochemical Kinetics, Noncovalent Interactions, Excited States, and Transition Elements: Two New Functionals and Systematic Testing of Four M06-Class Functionals and 12 Other Function. Theor. Chem. Acc..

[112] Grimme S., Antony J., Ehrlich S., Krieg H. (2010). A Consistent and Accurate Ab Initio Parametrization of Density Functional Dispersion Correction (DFT-D) for the 94 Elements H-Pu. J. Chem. Phys..

[113] Bartolotti L.J., Flurchick K. (2007). An Introduction to Density Functional Theory. Rev. Comput. Chem..

[114] Rampogu S., Baek A., Zeb A., Lee K.W. (2018). Exploration for Novel Inhibitors Showing Back-to-Front Approach against VEGFR-2 Kinase Domain (4AG8) Employing Molecular Docking Mechanism and Molecular Dynamics Simulations. BMC Cancer.

[115] Daina A., Michielin O., Zoete V. (2017). SwissADME: A Free Web Tool to Evaluate Pharmacokinetics, Drug-Likeness and Medicinal Chemistry Friendliness of Small Molecules. Sci. Rep..

[116] Daina A., Zoete V. (2016). A BOILED-Egg To Predict Gastrointestinal Absorption and Brain Penetration of Small Molecules. ChemMedChem.

[117] Fatima S., Gupta P., Sharma S., Sharma A., Agarwal S.M. (2019). ADMET Profiling of Geographically Diverse Phytochemical Using Chemoinformatic Tools. Future Med. Chem..

[118] Lipinski C.A. (2001). Drug-like Properties and the Causes of Poor Solubility and Poor Permeability. J. Pharmacol. Toxicol. Methods.

[119] Ghose A.K., Viswanadhan V.N., Wendoloski J.J. (1999). A Knowledge-Based Approach in Designing Combinatorial or Medicinal Chemistry Libraries for Drug Discovery. 1. A Qualitative and Quantitative Characterization of Known Drug Databases. J. Comb. Chem..

[120] Veber D.F., Johnson S.R., Cheng H., Smith B.R., Ward K.W., Kopple K.D. (2002). Molecular Properties That Influence the Oral Bioavailability of Drug Candidates. J. Med. Chem..

[121] Egan W.J., Merz K.M., Baldwin J.J. (2000). Prediction of Drug Absorption Using Multivariate Statistics. J. Med. Chem..

[122] Muegge I., Heald S.L., Brittelli D. (2001). Simple Selection Criteria for Drug-like Chemical Matter. J. Med. Chem..

[123] Martin Y.C. (2005). A Bioavailability Score. J. Med. Chem..

[124] Jasial S., Hu Y., Bajorath J. (2017). How Frequently Are Pan-Assay Interference Compounds Active? Large-Scale Analysis of Screening Data Reveals Diverse Activity Profiles, Low Global Hit Frequency, and Many Consistently Inactive Compounds. J. Med. Chem..

[125] Brenk R., Schipani A., James D., Krasowski A., Gilbert I.H., Frearson J., Wyatt P.G. (2008). Lessons Learnt from Assembling Screening Libraries for Drug Discovery for Neglected Diseases. ChemMedChem.

[126] Gomez L.A., Alekseev A.E., Aleksandrova L.A., Brady P.A., Terzic A. (1997). Use of the MTT Assay in Adult Ventricular Cardiomyocytes to Assess Viability: Effects of Adenosine and Potassium on Cellular Survival. J. Mol. Cell. Cardiol..

[127] van Meerloo J., Kaspers G.J.L., Cloos J., Cree I.A. (2011). Cell Sensitivity Assays: The MTT Assay. Cancer Cell Culture: Methods and Protocols.

[128] Louis K.S., Siegel A.C., Stoddart M.J. (2011). Cell Viability Analysis Using Trypan Blue: Manual and Automated Methods. Mammalian Cell Viability.

[129] Dawson C.W., Young L.S., Wilson J.B., May G.H.W. (2001). In Vitro Assays to Study Epithelial Cell Growth. Epstein-Barr Virus Protocols.

[130] Dong X., Xinglu Z., Jing H., Chen J., Liu T., Yang B., He Q., Yongzhou H. (2011). Pharmacophore Identification, Virtual Screening and Biological Evaluation of Prenylated Flavonoids Derivatives as PKB/Akt1 Inhibitors. Eur. J. Med. Chem..

[131] Thangapandian S., John S., Sakkiah S., Lee K.W. (2010). Ligand and Structure Based Pharmacophore Modeling to Facilitate Novel Histone Deacetylase 8 Inhibitor Design. Eur. J. Med. Chem..

