# Supplementary material for: Identification of Active Compounds against Melanoma Growth by Virtual Screening for Non-Classical Human DHFR Inhibitors
Source: Int J Mol Sci. 2022 Nov 11;23(22):13946. doi: 10.3390/ijms232213946 (PMC9694616; doi:10.3390/ijms232213946)
Supplement: Supplementary file 1 [file ijms-23-13946-s001.zip › ijms-1930418-supplementary.pdf]

Supplementary Material

Identification of active compounds against melanoma growth by virtual screening for non-classical HUMAN DHFR inhibitors

Andrés Felipe Vásquez[1,2], Luis Alberto Gómez[3,4], Andrés González-Barrios[1], and Diego Mauricio Riaño-Pachón[5*]

[1] Products and Processes Design Group (GDPP), School of Chemical Engineering, Universidad de los Andes, Bogotá, Colombia

[2] Naturalius S.A.S, Bogotá, Colombia

[3] Molecular Physiology Laboratory, Instituto Nacional de Salud, Bogotá, Colombia

[4] Department of Physiological Sciences, School of Medicine, Universidad Nacional de Colombia, Bogotá, Colombia.

[5] Center of Nuclear Energy in Agriculture (CENA), University of São Paulo (USP), Piracicaba, Brazil

[*]Corresponding author

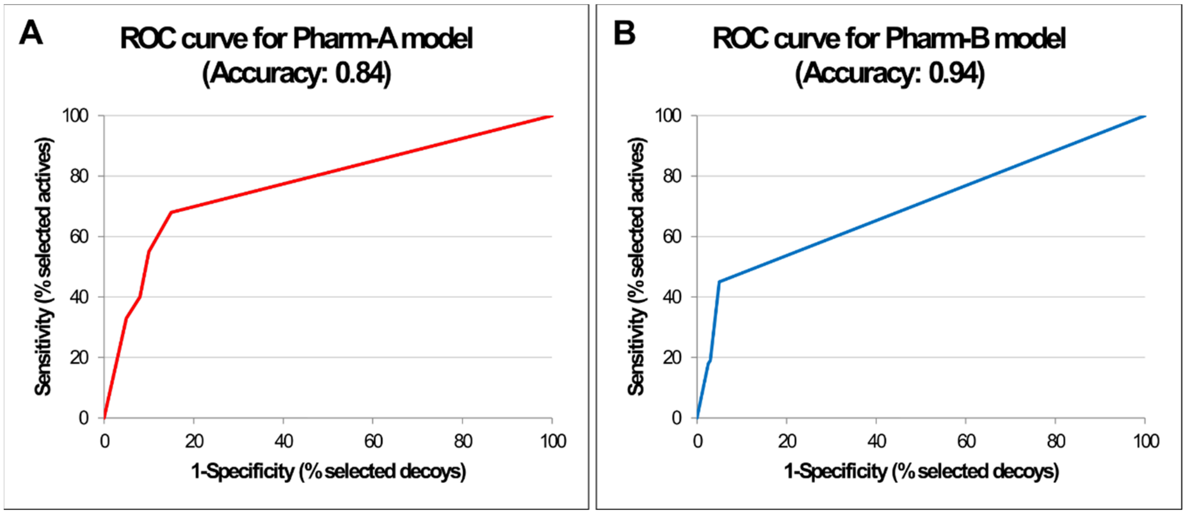

Figure S1. ROC curves of pharmacophore models generated in LigandScout. The performance of the pharmacophore models A) Pharm-A and B) Pharm-B was estimated by ROC curve analysis using well-known active and inactive compounds in a decoy set. The red and blue curves are the ROC curves for Pharm-A and Pharm-B, respectively. Accuracy was calculated according to the following formula: (TP+TN)/(TP+FP+TN+FN) [1].

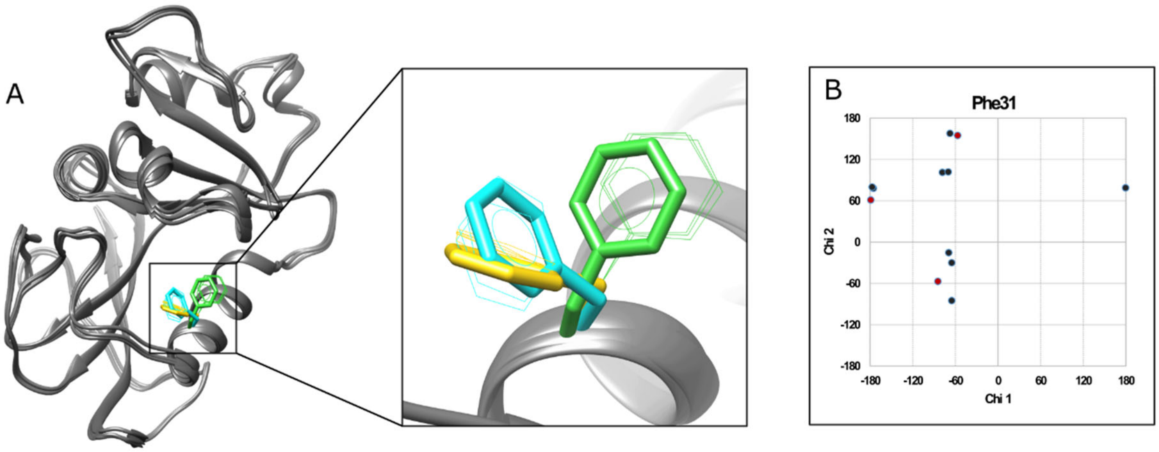

Figure S2. Structural alignment of hDHFR crystals selected for the conformational ensemble. A) Full and zoomed view of the three selected 3D structures (PDB codes:1KMV [2], 1U72 [3] and 3NXV [4]), emphasizing in the clustering of their Phe31 side-chains (stick representation). Dunbrack, Dynameomics and Richardson rotamers with highest probability (wire representation) are illustrated in gold, cyan and lime green, respectively. B) Janin plot describing the Phe31 dihedral angles Chi1 and Chi2. The combinations of these angles in the selected structures are illustrated as dark red points.

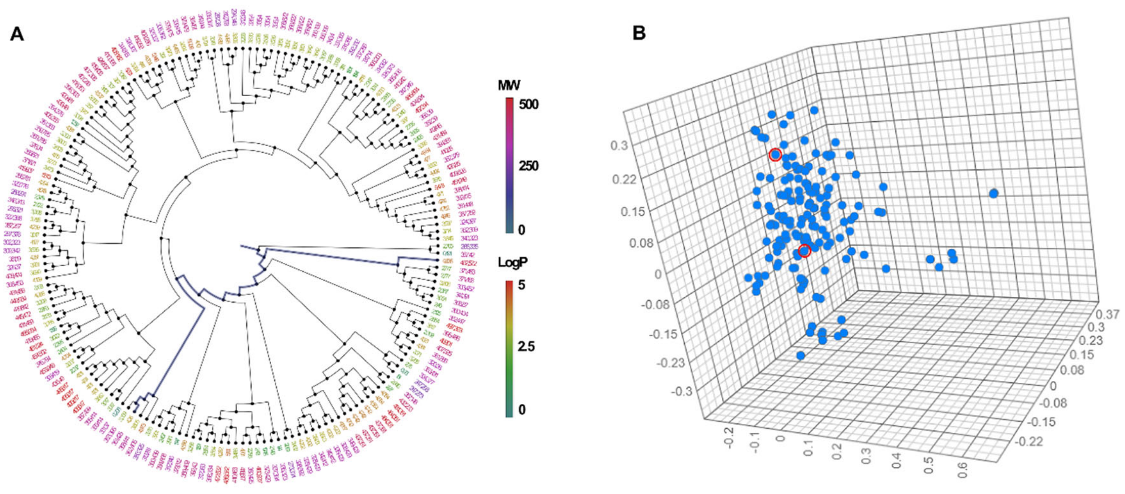

Figure S3. Hierarchical clustering and multidimensional scaling on hit dataset after virtual screening. Average hierarchical clustering (A) and multidimensional scaling (B) generated in ChemMine Tools based on pairwise ligand similarity for 173 ligands. The polar tree layout representing the ligands in our dataset was generated using the graphical software FigTree, and individual hit compounds at the end of each branch are labeled and colored by lipophilicity (logP; internal colored circle) and by molecular weight (MW; external colored circle). Hit compounds C1 and C2 are shown as shaded lines in (A) and as red-encircled spheres in (B).

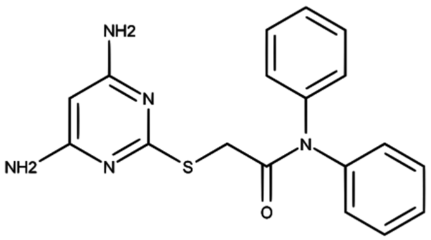

C1

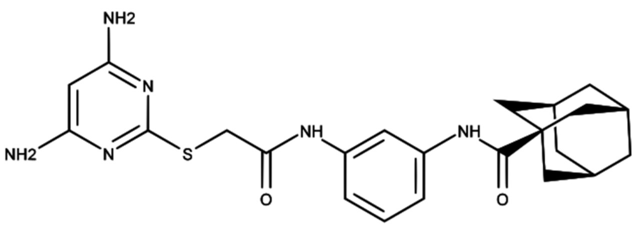

C2

Figure S4. Chemical structures of hit compounds C1 and C2. The 2D chemical structures of compounds C1 (ZINC00907702; 2-(4,6-diaminopyrimidine-1,3-diium-2-yl)sulfanyl-N,N-diphenylacetamide) and C2 (ZINC20102709; N-[3-[[2-(4,6-diaminopyrimidin-2-yl)sulfanylacetyl]amino]phenyl]adamantane-1-carboxamide), identified after virtual screening procedures, were both prepared with MarvinSketch v17.14, 2017, ChemAxon.

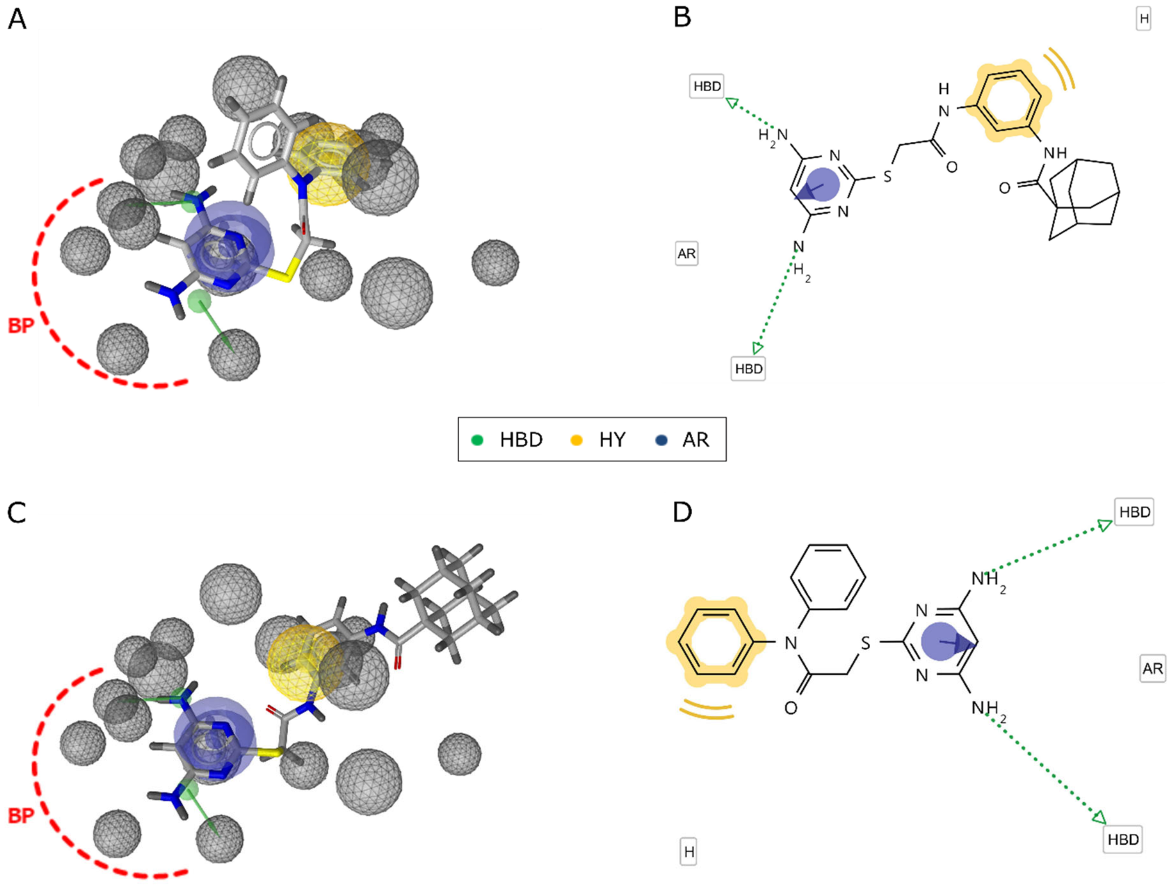

Figure S5. **Overlay of pharmacophore model Pharm-B with hits C1 and C2.** These hit compounds are showed aligned with PharmB model previously used in the screening in both 3D (shown here as (A) for C1 and (C) for C2) and 2D representations (shown here as (B) for C1 and (D) for C2), respectively. Hydrogen bond donor (HBD), hydrophobic group (HY), and aromatic ring (AR) features are colored green, gold and blue, respectively. Excluded volume spheres are illustrated in gray color.

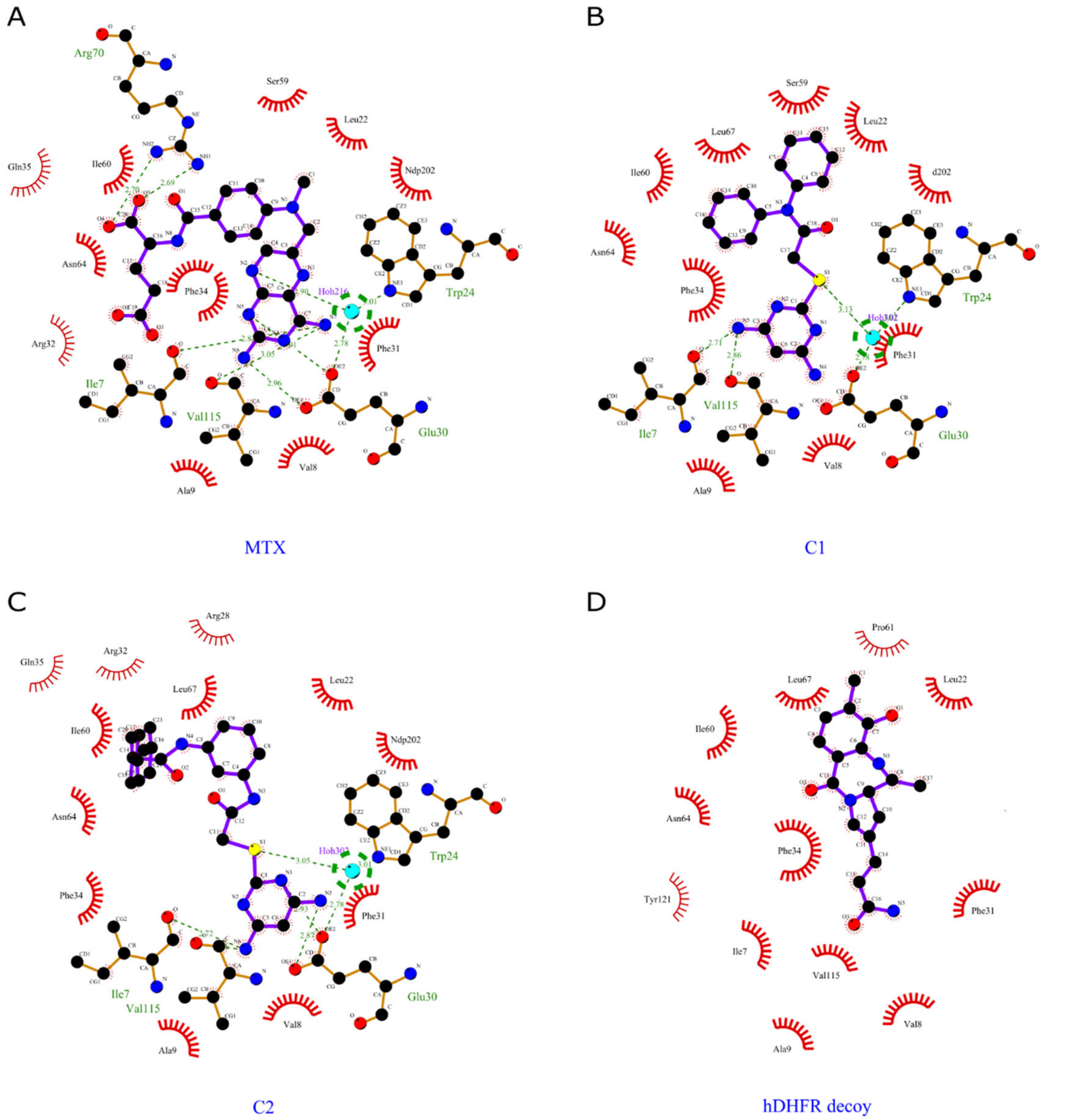

Figure S6. Docking pose analysis for hit compounds C1 and C2 including (and not including) water molecules. Superimposed 2D docked poses of MTX (A), hit compounds C1 (B) and C2 (C), and a selected DHFR decoy (D) generated by the program Ligplot+. H-bonds are shown as green dashed lines with their corresponding distances in angstroms (Å) and hydrophobic are indicated by spoked

arcs pointing towards each compound. Compounds C1 and C2 were predicted to interact with a conserved water molecule in human DHFR active site (inside of dashed line green circle), as observed also for MTX but not for the hDHFR decoy. A structurally conserved water molecule was predicted to facilitate the interaction with hDHFR active site residues Glu30 and Trp24.

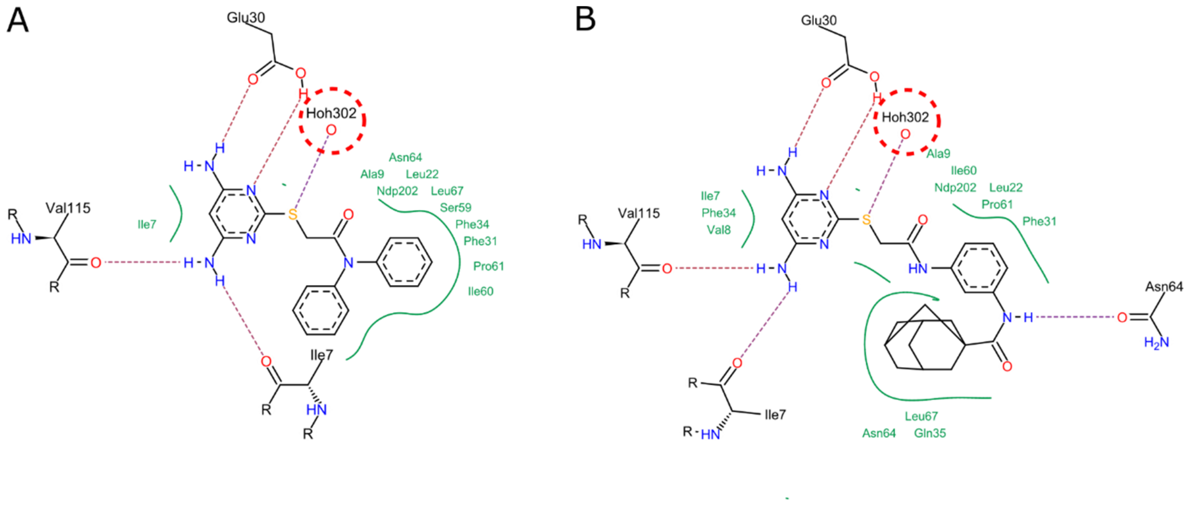

Figure S7. Docking pose analysis for hit compounds C1 and C2 by FlexX. 2D docked diagrams generated by the program FlexX and visualized by PoseView for either C1 (A) and C2 (B) bound into hDHFR active site. Both compounds were predicted to interact with a conserved water molecule in human DHFR active site (inside of a dashed line red circle in each case). H-bonds are indicated here as dashed lines, and hydrophobic interactions are shown as green solid lines.

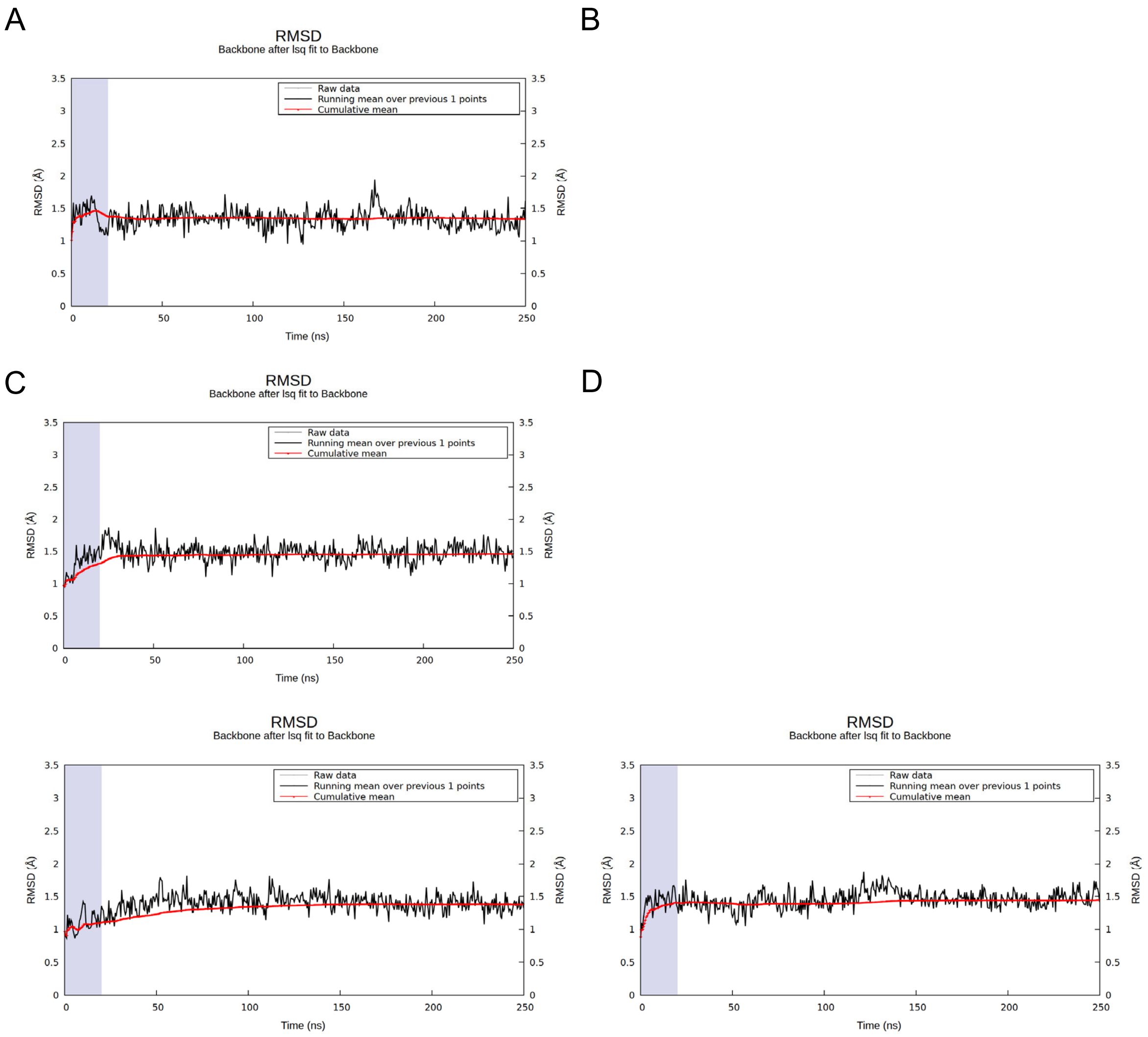
A
B
C
D
RMSD
Backbone after lsq fit to Backbone
Raw data
Running mean over previous 1 points
Cumulative mean
RMSD (Å)
Time (ns)
0
0.5
1
1.5
2
2.5
3
3.5
50
100
150
200
250

Figure S8. RMSD plot of the backbone atoms of hDHFR. The RMSD calculation was performed on the Cα atoms of hDHFR over 250 ns of MD simulations for the a) unbound form, and the bound form complexed with b) MTX, c) C1 and d) C2. The gray boxes in each graph represent equilibration time and, hence, this period was not considered for further analysis.

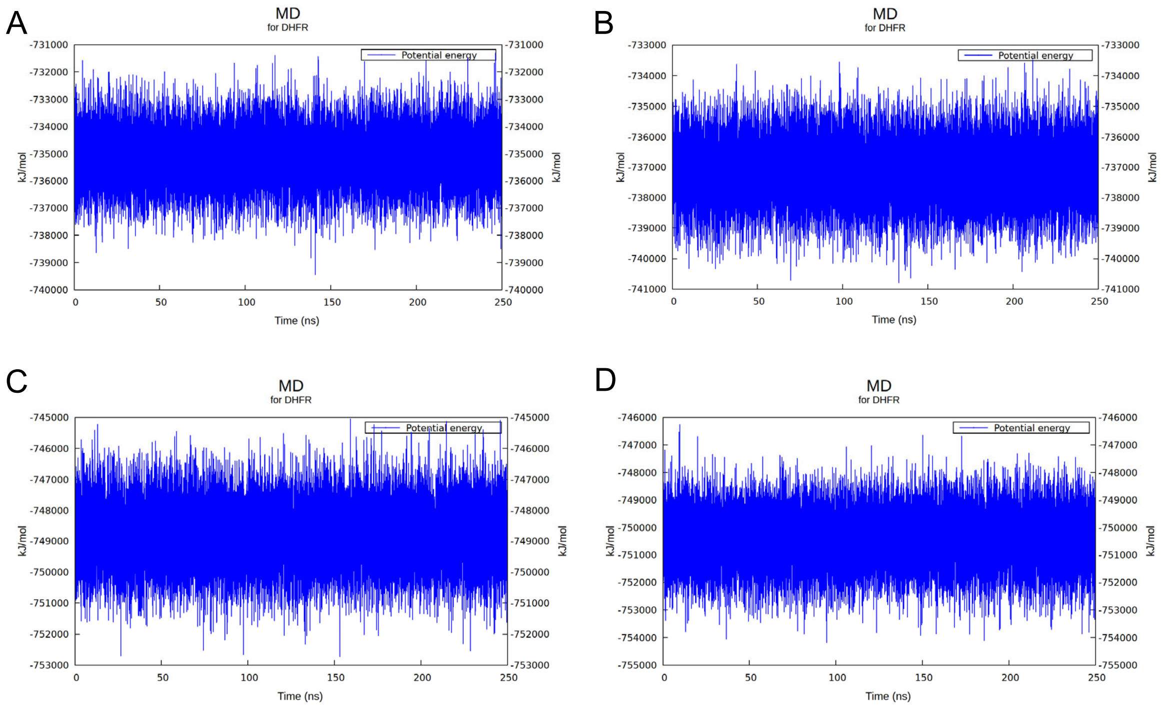

Figure S9. Potential energy plot obtained from the MD simulation for hDHFR. Calculation of potential energy fluctuation curves was performed on the atoms of enzymes hDHFR over 250 ns of MD simulations for the unbound form (A) and the bound form complexed with active compound MTX (B) and the hit compounds C1 (C) and C2 (D).

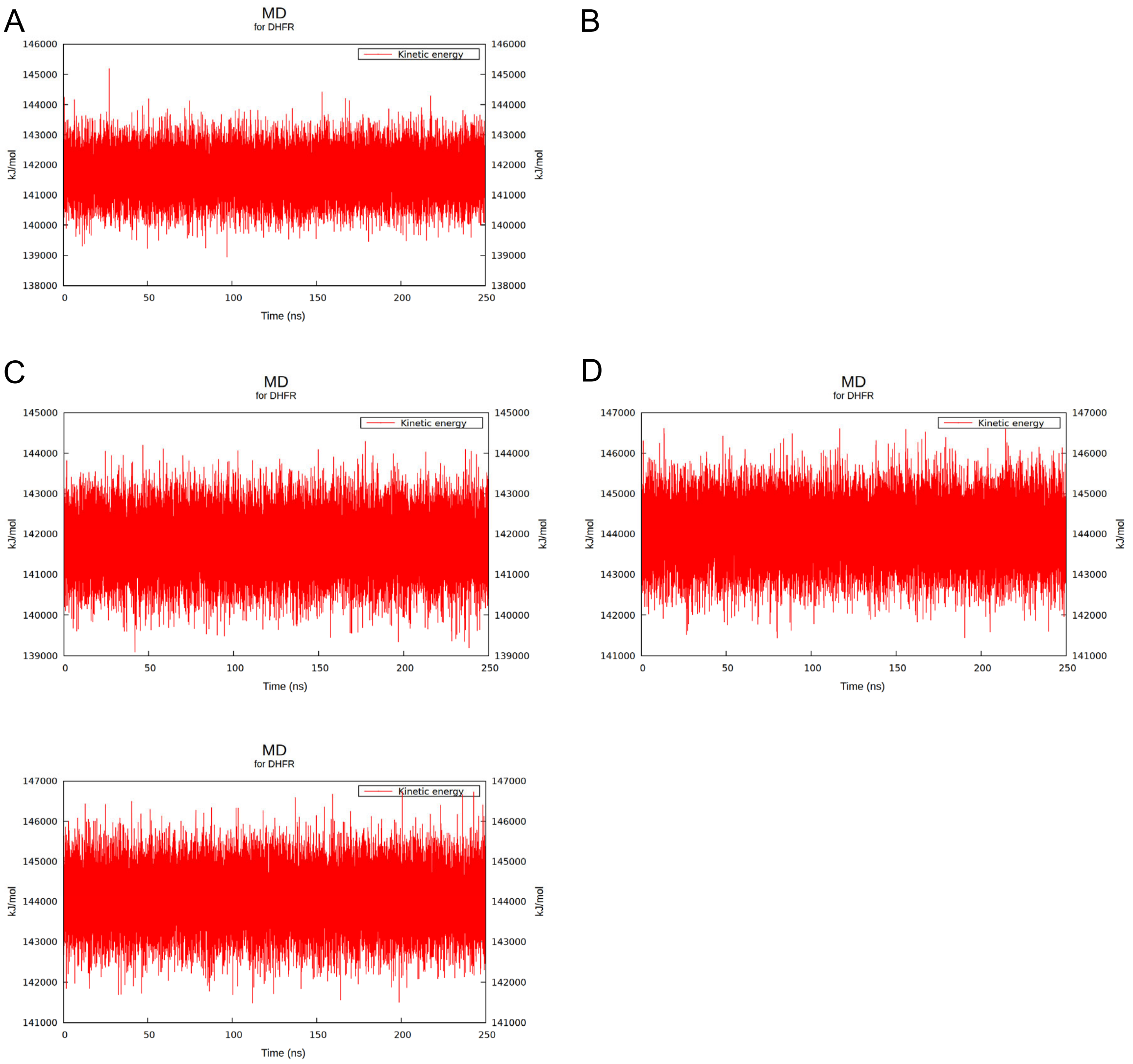

Figure S10. Kinetic energy plot obtained from the MD simulation for hDHFR. Calculation of kinetic energy fluctuation curves was performed on the atoms of enzymes hDHFR over 250 ns of MD simulations for the unbound form (A) and the bound form complexed with active compound MTX (B) and the hit compounds C1 (C) and C2 (D).

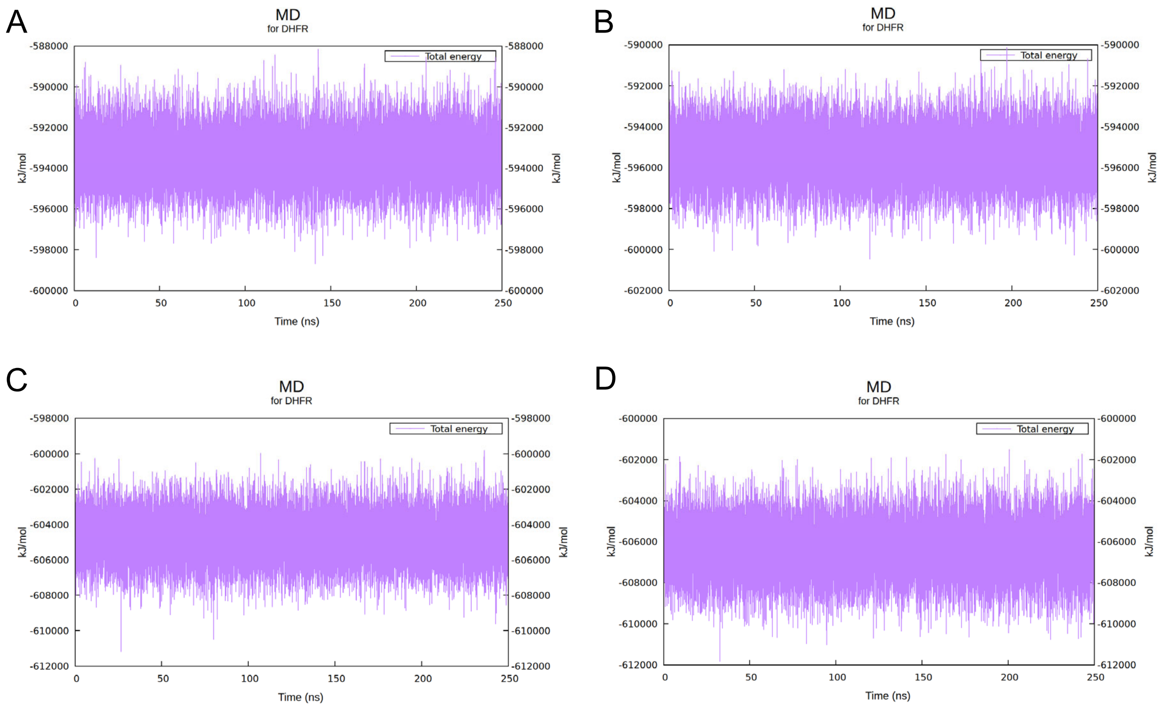

Figure S11. Total energy plot obtained from the MD simulation for hDHFR. Calculation of total energy fluctuation curves was performed on the atoms of enzymes hDHFR over 250 ns of MD simulations for the unbound form (A) and the bound form complexed with active compound MTX (B) and the hit compounds C1 (C) and C2 (D).

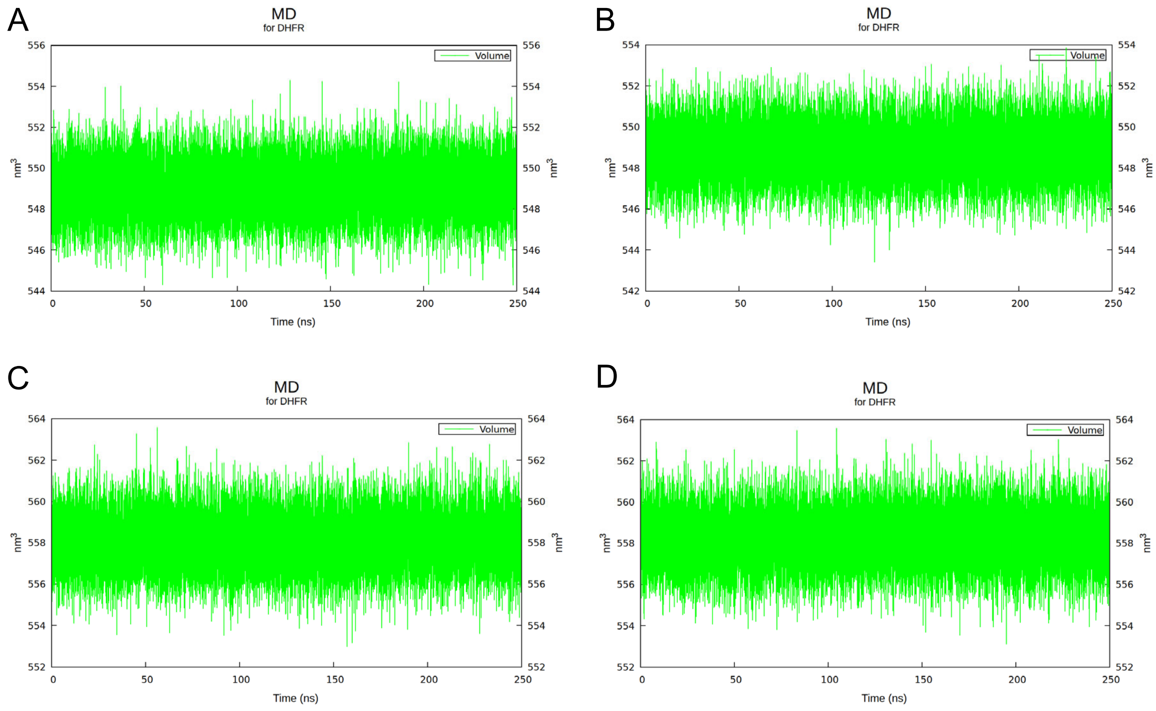

Figure S12. Volume plot obtained from the MD simulation for hDHFR. Calculation of volume fluctuation curves was performed on the atoms of enzymes hDHFR over 250 ns of MD simulations for the unbound form (A) and the bound form complexed with active compound MTX (B) and the hit compounds C1 (C) and C2 (D).

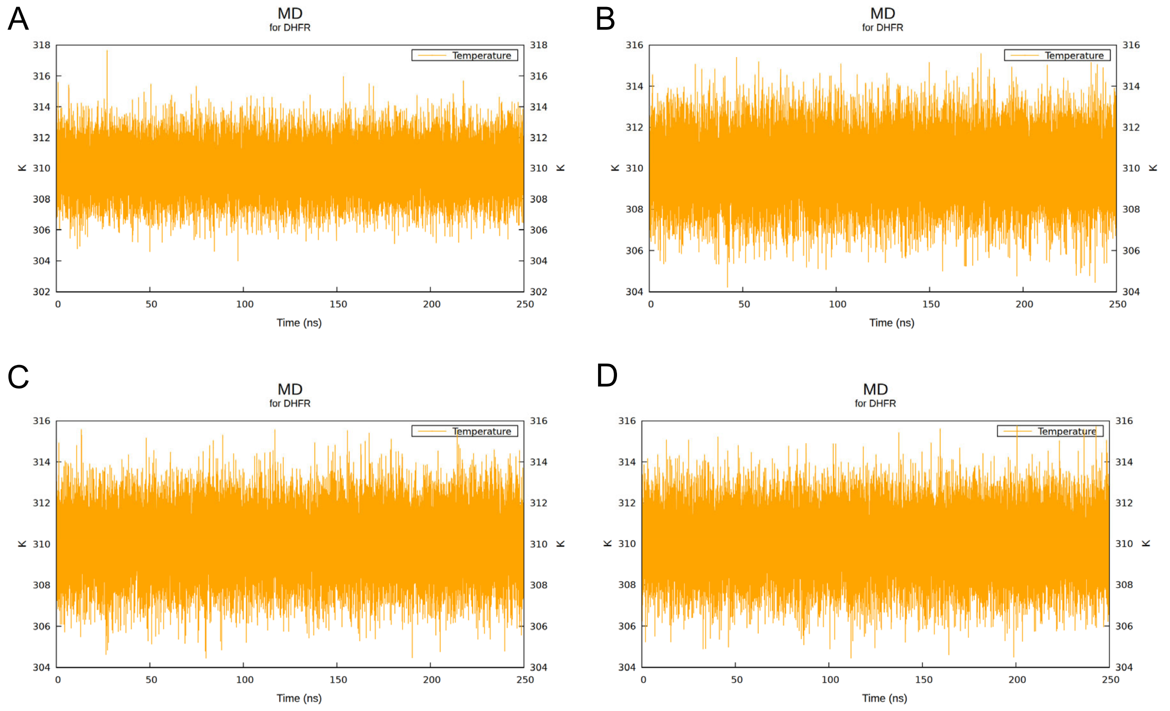

Figure S13. Temperature plot obtained from the MD simulation for hDHFR. Calculation of temperature fluctuation curves was performed on the atoms of enzymes hDHFR over 250 ns of MD simulations for the unbound form (A) and the bound form complexed with active compound MTX (B) and the hit compounds C1 (C) and C2 (D).

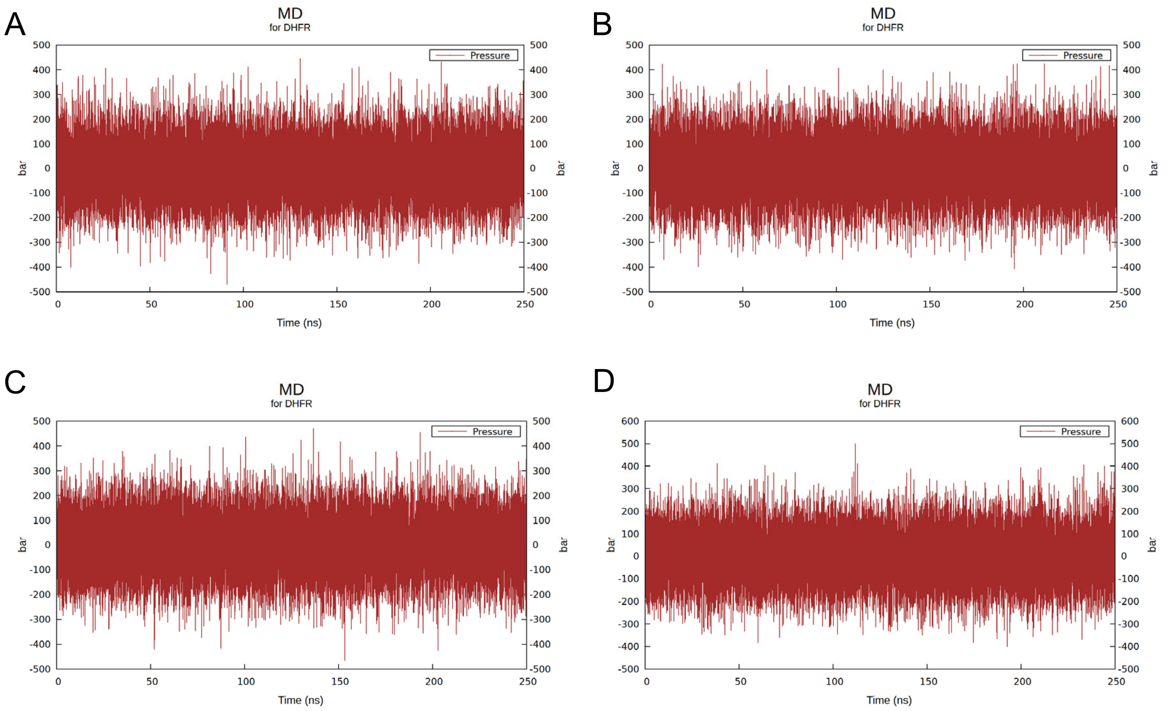

Figure S14. Pressure plot obtained from the MD simulation for hDHFR. Calculation of pressure fluctuation curves was performed on the atoms of enzymes hDHFR over 250 ns of MD simulations for the unbound form (A) and the bound form complexed with active compound MTX (B) and the hit compounds C1 (C) and C2 (D).

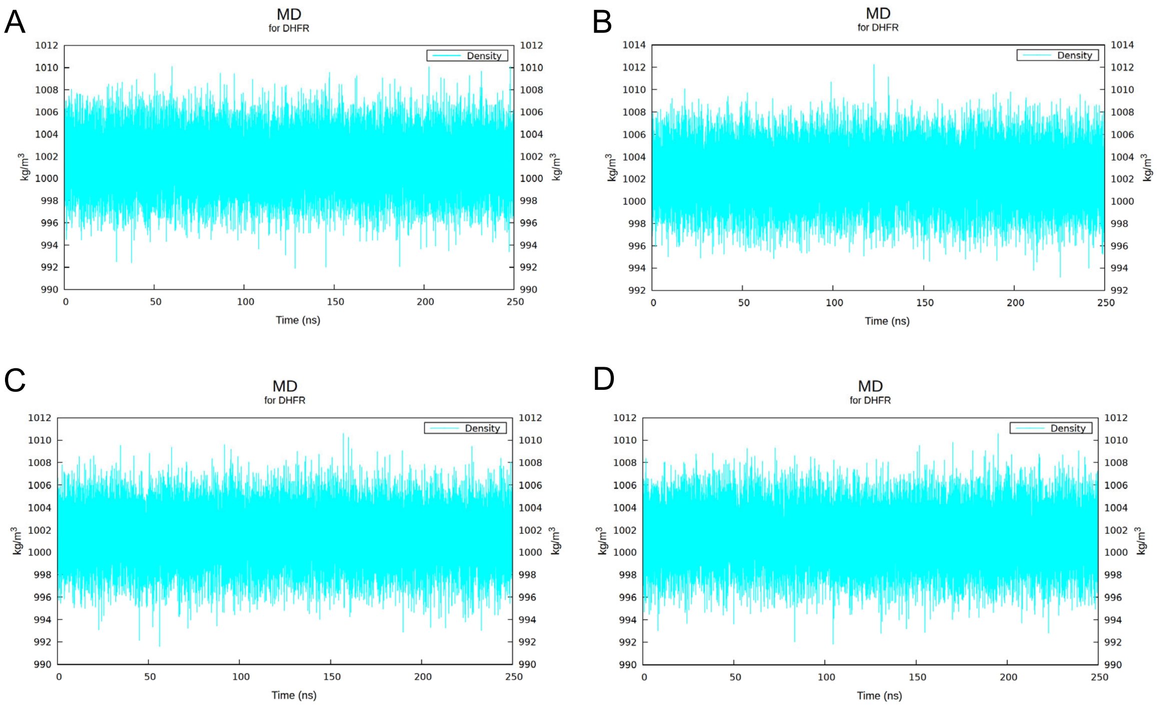

Figure S15. Density plot obtained from the MD simulation for hDHFR. Calculation of density fluctuation curves was performed on the atoms of enzymes hDHFR over 250 ns of MD simulations for the unbound form (A) and the bound form complexed with active compound MTX (B) and the hit compounds C1 (C) and C2 (D).

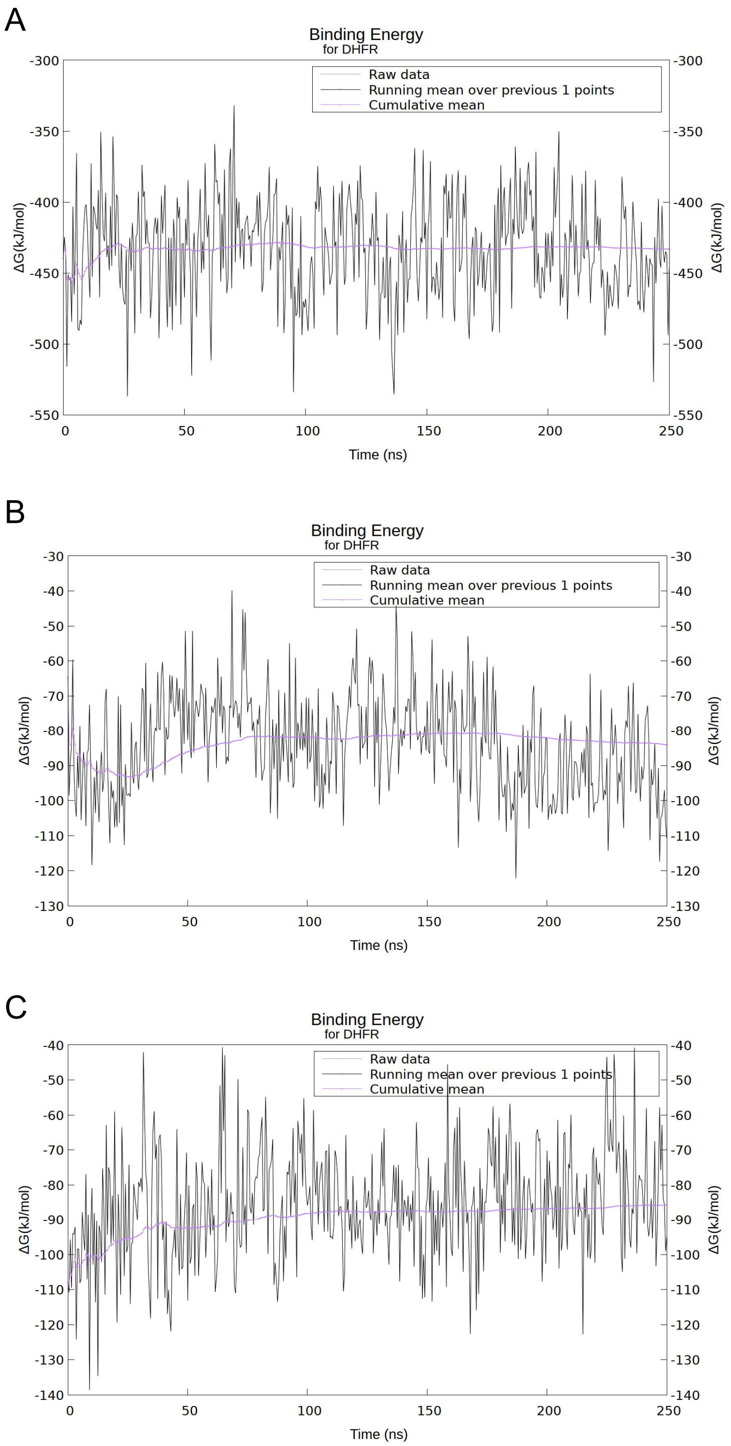

Figure S16. Binding energy plot for hDHFR. Binding energy calculation was carried out for hDHFR over 250 ns of MD simulations in complex with reference compound MTX (A), C1 (B), and C2 (C).

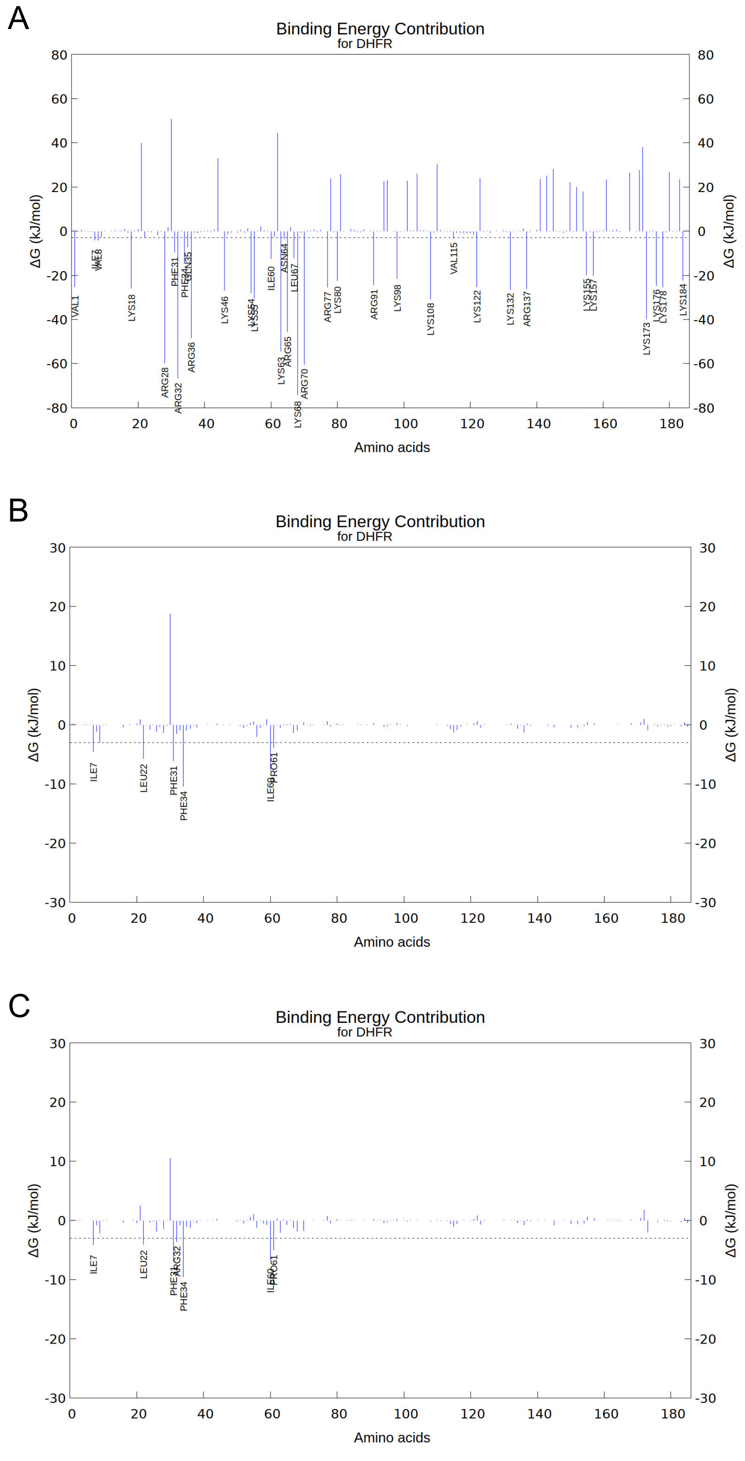

Figure S17. Contribution of each residue of hDHFR to ligand binding. Contribution from each residue was estimated for hDHFR over 250 ns of MD

simulations complexed with MTX (A), C1 (B) and C2 (C). The horizontal discontinuous lines in each graph represent an energy value equal to -3 kJ/mol.

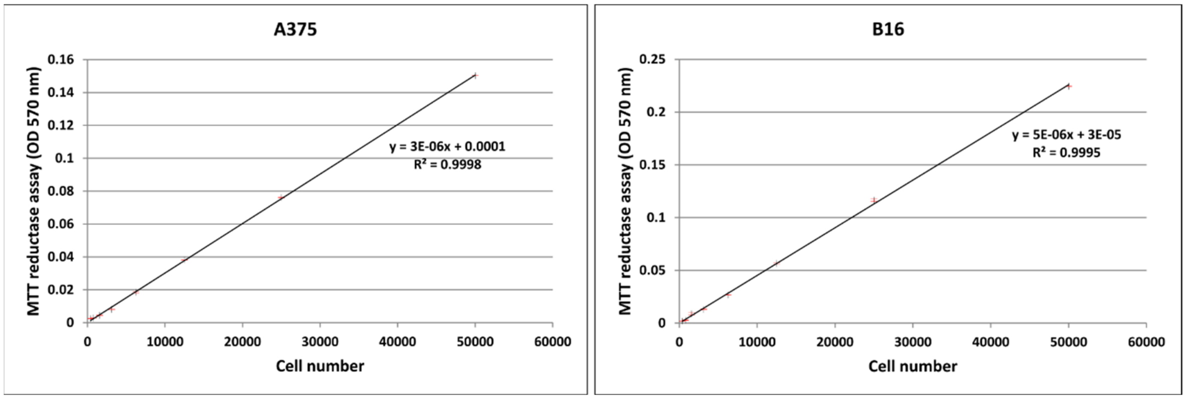

Figure S18. Calibration curves for dose-dependent assays. Absorbance versus cell number for human (A375; *left panel*), and mouse (B16; *right panel*) melanoma cell lines is plotted. The number of cells is directly proportional to the level of the formazan product generated (R^2=0.9998 and R^2=0.9995 for A375 and B16 melanoma cells, respectively) after quantification at 570nm.

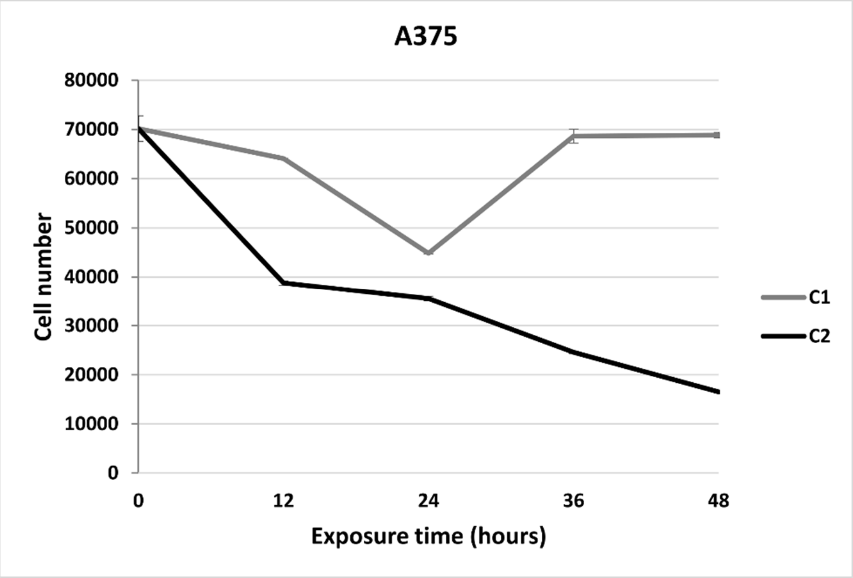

Figure S19. Time-dependent effect of compounds C1 and C2 on human melanoma cell line A735. Cell viability determined by MTT reductase activity after exposure to hit compounds C1 (*gray-colored line*) C2 (*black-colored line*) during a period of 12, 24 36 and 48 hours, respectively. The concentration used for each compound was 11 μg/mL.

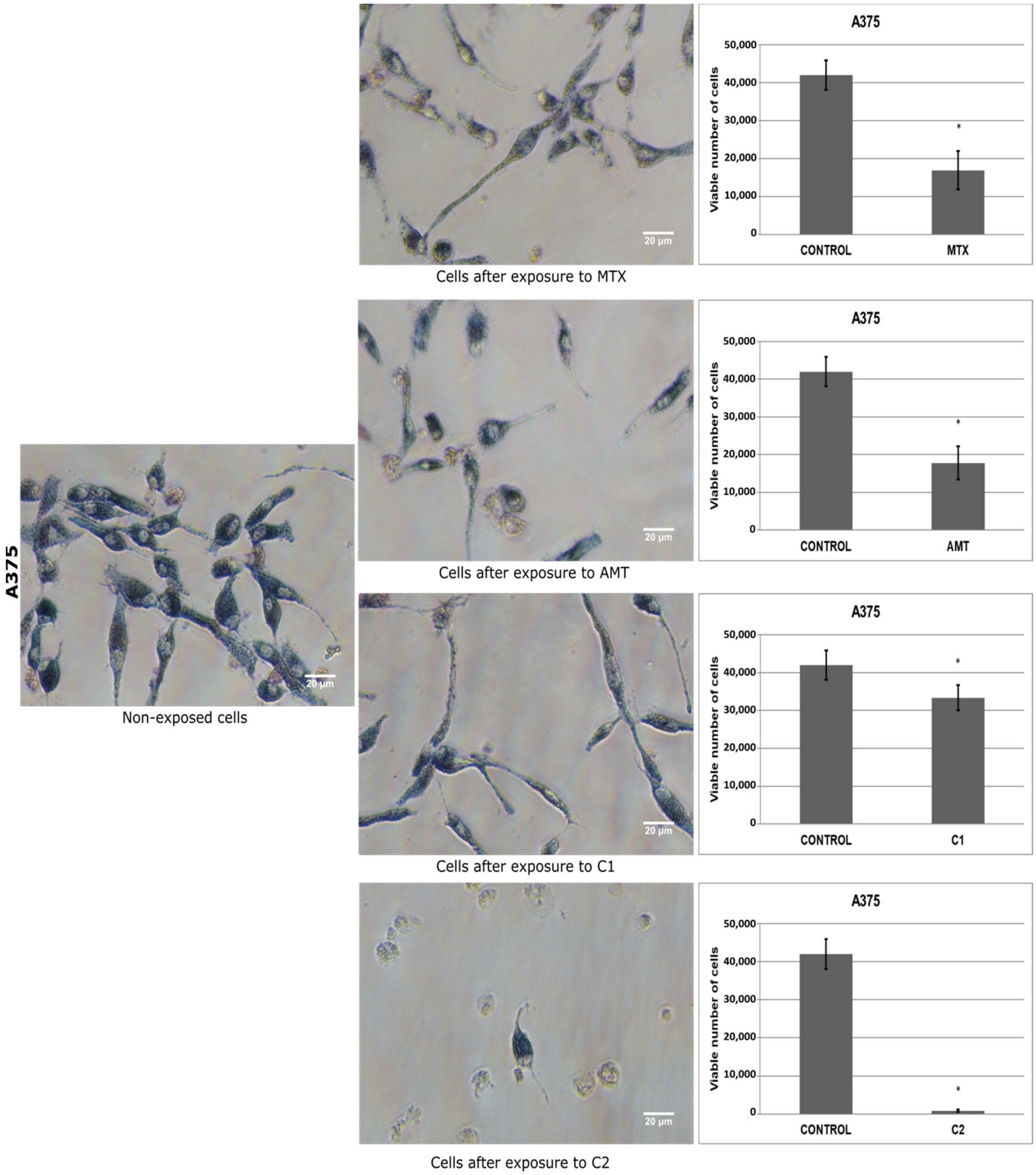

Figure S20. **Effect on A375 cells after exposure to C1 and C2 compared to MTX and AMT**. Human (A375) melanoma cells were seeded into a 48-well plate overnight and cell viability was determined by MTT reductase activity and trypan blue (TB) exclusion assay after exposure to hit compounds C1 and C2, as well as reference

compounds MTX and AMT, at a concentration of 11 μg/mL during a period of 48 hours. A moderate reduction on cell viability was estimated for human melanoma cells after exposure to hit compound C1, but to a lesser extent compared to that observed for MTX and AMT. Conversely, a remarkable reduction on cell viability after exposure to hit compound C2 was estimated, which was higher than that observed for reference compounds (see text for details).

PDB ID	Ile7	Val8	Ala9	Leu22	Glu30	Phe31	Phe34	Pro61	Val115	Thr136
1DHF	◎	◎	◎		◎	◎	◎		◎	◎
1DRF	◎	◎	◎		◎	◎	◎	◎	◎	◎
1HFR	◎	◎	◎	◎	◎	◎	◎		◎	◎
1KMS	◎	◎	◎	◎	◎	◎	◎	◎	◎	◎
1KMV	◎	◎	◎	◎	◎	◎	◎	◎	◎	◎
1MVS	◎	◎	◎		◎	◎	◎	◎	◎	◎
1MVT	◎	◎	◎	◎	◎	◎	◎		◎	◎
1OHJ	◎	◎	◎		◎	◎	◎		◎	
1OHK	◎	◎	◎	◎	◎	◎	◎	◎	◎	◎
1PD8	◎	◎	◎	◎	◎	◎	◎	◎	◎	
1PD9	◎	◎	◎	◎	◎	◎	◎	◎	◎	
1S3U	◎	◎	◎	◎	◎	◎	◎	◎	◎	◎
1S3V	◎	◎	◎	◎	◎	◎	◎	◎	◎	◎
1S3W	◎	◎	◎	◎	◎	◎	◎	◎	◎	◎
1U72	◎	◎	◎	◎	◎	◎	◎		◎	
2C2S	◎	◎	◎	◎	◎		◎		◎	◎
2C2T	◎	◎	◎	◎	◎		◎	◎	◎	◎
2DHF	◎	◎	◎	◎	◎	◎	◎	◎	◎	◎
2W3A	◎	◎	◎	◎	◎	◎	◎	◎	◎	◎
2W3B	◎	◎	◎	◎	◎	◎	◎		◎	◎
2W3M	◎	◎	◎	◎	◎	◎	◎		◎	◎
3FS6	◎	◎	◎		◎	◎	◎		◎	◎
3GHW	◎	◎	◎	◎	◎	◎	◎	◎	◎	◎
3GYF	◎	◎	◎		◎	◎	◎		◎	◎
3NTZ	◎	◎	◎	◎	◎	◎	◎	◎	◎	◎
3NU0	◎	◎	◎	◎	◎	◎	◎		◎	◎
3NXR	◎	◎	◎	◎	◎	◎	◎	◎	◎	◎

**3NXT**	◎	◎	◎	◎	◎	◎	◎		◎	◎
**3NXV**	◎	◎	◎	◎	◎	◎	◎		◎	◎
**3NXX**	◎	◎	◎	◎	◎	◎	◎		◎	◎
**3NXY**	◎	◎	◎	◎	◎	◎	◎	◎	◎	◎
**3NZD**	◎	◎	◎	◎	◎	◎	◎	◎	◎	◎
**3S7A**	◎	◎	◎	◎	◎	◎	◎	◎	◎	◎
**4DDR**	◎	◎	◎	◎	◎	◎	◎	◎	◎	◎
**4G95**	◎	◎	◎	◎	◎	◎	◎	◎	◎	◎
**4KAK**	◎	◎	◎		◎	◎	◎	◎	◎	
**4KBN**	◎	◎	◎	◎	◎	◎	◎	◎	◎	◎
**4KD7**	◎	◎	◎	◎	◎	◎	◎	◎	◎	
**4KEB**	◎	◎	◎	◎	◎	◎	◎	◎	◎	◎
**4KFJ**	◎	◎	◎	◎	◎	◎	◎	◎	◎	◎
**4M6K**	◎	◎	◎	◎	◎	◎	◎		◎	◎
**4M6L**	◎	◎	◎	◎	◎	◎	◎	◎	◎	◎
**4QHV**	◎	◎	◎	◎	◎	◎	◎	◎	◎	◎
**4QJC**	◎	◎	◎	◎	◎		◎	◎	◎	◎
**5HPB**	◎	◎	◎	◎	◎	◎	◎	◎	◎	◎
**5HQY**	◎	◎	◎	◎	◎	◎	◎	◎	◎	◎
**5HQZ**	◎	◎	◎	◎	◎	◎	◎	◎	◎	◎
**5HSR**	◎	◎	◎	◎	◎	◎	◎		◎	◎
**5HSU**	◎	◎	◎	◎	◎	◎	◎	◎	◎	◎
**5HT4**	◎	◎	◎	◎	◎	◎	◎	◎	◎	◎
**5HT5**	◎	◎	◎	◎	◎	◎	◎	◎	◎	◎
**5HUI**	◎	◎	◎	◎	◎	◎	◎	◎	◎	◎
**5HVB**	◎	◎	◎	◎	◎	◎	◎	◎	◎	◎
**5HVE**	◎	◎	◎	◎	◎	◎	◎	◎	◎	◎

Table S1. Analysis of critical amino acid residues for hDHFR inhibition. A total of 54 native co-crystal structures in Protein Data Bank was analyzed in order to determine the amino acids interacting with different DHFR inhibitors. Any marked amino acid is an indication of its role in protein-ligand interaction in the corresponding PDB structure.

**Parameter**	**Values**	
	**Pharm-A**	**Pharm-B**
**Molecules in database (D)**	8204	8204
**Total actives in database (A)**	60	60
**True positives (TP)**	40	36
**True negatives (TN)**	6874	7680
**False positives (FP)**	1270	464
**False negatives (FN)**	20	24
**AUC 1%** [a]	0.13	0.79
**AUC 5%**	0.78	0.95
**AUC 10%**	0.87	0.95
**AUC 100%**	0.78	0.70
**EF 1%** [b]	1.70	13.30
**EF 5%**	6.70	8.00
**EF 10%**	5.50	7.40
**EF 100%**	4.30	7.40
**Sensitivity** [c]	0.67	0.60
**Specificity** [d]	0.84	0.94

Table S2. Statistical parameters from screening compounds in decoy set. [a] Area under ROC curve: $\sum_{x=2}^{N} TPR(x)[FPR(x) - FPR(x-1)]$ [5]; [b] Enrichment factor: [(TP*D)/((TP+FP)*A))] [6]; [c] Sensitivity, also called Recall or True Positive rate (TPR): [TP/(TP+FN)] [5]; [d] Specificity, also called True Negative Rate (TNR): [TN/(TN+FP)] [5].

Water molecule inclusion	Affinity Measurement	C1	C2	MTX	hDHFR decoy
YES	Binding energy (kcal/mol)	-9.73	-10.78	-12.95	-8.53
	Ki (nM)	73.94	50.13	0.32	560.64
NO	Binding energy (kcal/mol)	-9.95	-10.95	-12.36	-8.45
	Ki (nM)	51.17	15.9	0.87	643.11

Table S3. Predicted binding energy and Ki among hit compounds C1, C2, MTX and a hDHFR decoy. Binding energy was measured in kcal/mol and inhibition constant (Ki) values were calculated in nanomolar (nM) scale.

Ligand	MM/PBSA ΔG bind (kcal/mol)	HOMO (eV)	LUMO (eV)	Energy gap (eV)
**MTX**	-103.432 +/- 0.368	-0.784	4.339	5.123
**C1**	-20.068 +/- 0.148	-6.690	1.052	7.742
**C2**	-20.480 +/- 0.161	-6.602	1.290	7.892

Table S4. Results of MM/PBSA and electronic parameters calculated for active compound MTX and hit compounds C1 and C2. Binding energy was measured in

kJ/mol and HOMO, LUMO and energy gap values were all estimated in electron volts (eV).

Properties		MTX	C1	C2
**Pharmacokinetics**	**GI absorption**	Low	High	Low
	**BBB permeant**	No	No	No
	**P-gp substrate**	Yes	No	Yes
	**CYP1A2 inhibitor**	No	Yes	No
	**CYP2C9 inhibitor**	No	Yes	No
	**CYP3A4 inhibitor**	No	Yes	Yes
**Drug-likeness**	**Lipinski**	Yes	Yes	Yes
	**Ghose**	Yes	Yes	Yes
	**Veber**	No	Yes	No
	**Egan**	No	Yes	No
	**Muegge**	No	Yes	No
	**Bioavailability score**	0.11	0.55	0.55
**Medicinal Chemistry**	**PAINS alerts**	No	No	No
	**Brenk alerts**	No	No	No
	**Synthetic accesibility**	3.58	2.90	5.57

Table S5. ADME properties of the active compound MTX and the hit compounds C1 and C2 identified after the VS procedures. The different properties associated with pharmacokinetics, drug-likeness, and medicinal chemistry were estimated using the web-server SwissADME [7].

PDB ID	Resolution (Å)	Ligand
**1KMV**	1.05	LII
**1KMS**	1.09	LIH
**5HSR**	1.21	63Y
**3FS6**	1.23	DH1
**3GHW**	1.24	GHW
**2W3B**	1.27	VG9
**3NTZ**	1.35	3TZ
**3NU0**	1.35	3TU
**3NXR**	1.35	D2D
**3NXX**	1.35	D2D

Table S6. Top 10 highest resolution hDHFR crystal structures. Every hDHFR 3D structure is reported in terms of its corresponding 4-character unique PDB ID, resolution (Å) and its co-crystallized ligand.

References

1. Rizzi A, Fioni A. Virtual screening using PLS discriminant analysis and ROC curve approach: an application study on PDE4 inhibitors. *J. Chem. Inf. Model.* 48(8), 1686–92 (2008).

2. Klon AE, Héroux A, Ross LJ, *et al.* Atomic structures of human dihydrofolate reductase complexed with NADPH and two lipophilic antifolates at 1.09 Å and 1.05 Å resolution. *J. Mol. Biol.* 320(3), 677–693 (2002).

3. Cody V, Luft JR, Pangborn W. Understanding the role of Leu22 variants in methotrexate resistance: comparison of wild-type and Leu22Arg variant mouse and human dihydrofolate reductase ternary crystal complexes with methotrexate and NADPH. *Acta Crystallogr. D. Biol. Crystallogr.* 61(Pt 2), 147–55 (2005).

4. Cody V, Piraino J, Pace J, Li W, Gangjee A. Preferential selection of isomer binding from chiral mixtures: alternate binding modes observed for the E and Z isomers of a series of 5-substituted 2,4-diaminofuro[2,3-d]pyrimidines as ternary complexes with NADPH and human dihydrofolate reductase. *Acta Crystallogr. Sect. D Biol. Crystallogr.* 66(Pt 12), 1271–1277 (2010).

5. Dong X, Xinglu Z, Jing H, *et al.* Pharmacophore identification, virtual screening and biological evaluation of prenylated flavonoids derivatives as PKB/Akt1 inhibitors. *Eur. J. Med. Chem.* 46(12), 5949–5958 (2011).

6. Thangapandian S, John S, Sakkiah S, Lee KW. Ligand and structure based pharmacophore modeling to facilitate novel histone deacetylase 8 inhibitor design. *Eur. J. Med. Chem.* 45(10), 4409–4417 (2010).

7. Daina A, Michielin O, Zoete V. SwissADME: A free web tool to evaluate pharmacokinetics, drug-likeness and medicinal chemistry friendliness of small molecules. *Sci. Rep.* 7(March), 1–13 (2017).
